# Adiposity and risks of gastrointestinal cancers: A 10‐year prospective study of 0.5 million Chinese adults

**DOI:** 10.1002/ijc.35303

**Published:** 2024-12-31

**Authors:** Wing Ching Chan, Iona Millwood, Christiana Kartsonaki, Huaidong Du, Daniel Schmidt, Rebecca Stevens, Junshi Chen, Pei Pei, Canqing Yu, Dianjianyi Sun, Jun Lv, Xianyong Han, Liming Li, Zhengming Chen, Ling Yang, for the China Kadoorie Biobank (CKB) Collaborative Group

**Affiliations:** ^1^ Clinical Trial Service Unit and Epidemiological Studies Unit (CTSU), Nuffield Department of Population Health University of Oxford Oxford UK; ^2^ Medical Research Council Population Health Research Unit (MRC PHRU), Nuffield Department of Population Health University of Oxford Oxford UK; ^3^ China National Center For Food Safety Risk Assessment Beijing China; ^4^ Peking University Center for Public Health and Epidemic Preparedness & Response Beijing China; ^5^ Department of Epidemiology and Biostatistics, School of Public Health Peking University Health Science Center Beijing China; ^6^ Key Laboratory of Epidemiology of Major Diseases (Peking University), Ministry of Education Beijing China; ^7^ Yongqinglu Community Health Service Qingdao China

**Keywords:** adiposity, body fat, gastrointestinal cancers

## Abstract

Associations of adiposity with risks of oesophageal squamous cell carcinoma (ESCC) and non‐cardia stomach cancer, both prevalent in China, are still inconclusive. While adiposity is an established risk factor for colorectal cancer, the relevance of fat‐free mass and early‐adulthood adiposity remains to be explored. The prospective China Kadoorie Biobank study included 0.5 million adults (aged 30–79 years) from 10 areas in China. Participants' body size and composition were measured at baseline and at resurveys (amongst a subset). After >10 years of follow‐up, 2350, 3345 and 3059 incident cases of oesophageal (EC), stomach (SC) and colorectal (CRC) cancers were recorded, respectively. Cox regression was used to estimate hazard ratios (HRs) for these cancers in relation to different adiposity traits. General and central adiposity were inversely associated with EC (primarily ESCC) risk, with HRs of 0.81 (95% CI 0.77–0.85), 0.76 (0.72–0.81) and 0.87 (0.83–0.92) per SD increase in usual levels of BMI, body fat percentage (BF%) and waist circumference (WC), respectively. Adiposity was also inversely associated with SC risk [HR = 0.79 (0.75–0.83) and 0.88 (0.84–0.92) per SD increase in usual BF% and WC], with heterogeneity by cardia and non‐cardia subsites, and positively associated with CRC [HR = 1.09 (1.03–1.15) and 1.17 (1.12–1.22) per SD higher usual BF% and WC]. Fat‐free mass was inversely associated with EC [HR = 0.93 (0.89–0.98) per SD increase] but positively associated with CRC [1.09 (1.04–1.14)], while BMI at age 25 was positively associated with all three cancers. After mutual adjustment, general adiposity remained inversely associated with EC and SC, while central adiposity remained positively associated with CRC.

## Graphical abstract

What's New

While obesity is an established risk factor for several types of gastrointestinal cancers, its relationships with oesophageal and stomach cancers are unclear. This prospective study of adiposity with oesophageal (primarily squamous cell carcinoma), stomach (primarily non‐cardia) and colorectal cancer risks assessed multiple bioimpedance and anthropometric traits. General adiposity was inversely associated with oesophageal and stomach (overall and non‐cardia) cancer risks; associations with central adiposity were less clear. The authors also provided new evidence for fat‐free mass, with a suggestive inverse association with oesophageal cancer and a positive association with colorectal cancer.

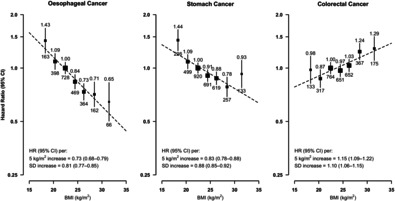

## 1 INTRODUCTION

Oesophageal, stomach and colorectal cancers are major gastrointestinal (GI) cancers responsible for over 3.6 million new cancer cases and 2.2 million deaths worldwide in 2020.^1^, ^2^ China alone accounted for over half of the global burden of oesophageal (EC) and stomach cancer (SC),^3^, ^4^ and unlike many Western countries where the predominant histological subtype of EC is adenocarcinoma (EAC), >90% of EC cases in China are oesophageal squamous cell carcinoma (ESCC).^1^, ^4^, ^5^

Obesity was categorised in the latest World Cancer Research Fund (WCRF) report as a ‘convincing’ cause of EAC and colorectal cancer (CRC), and a ‘probable’ cause of cardia SC, while evidence was judged to be too limited for ESCC and non‐cardia SC.^6^, ^7^, ^8^ These conclusions, however, were based primarily on evidence from ‘traditional’ anthropometric measures such as body mass index (BMI), waist circumference (WC) and waist‐to‐hip ratio (WHR).^6^, ^7^, ^8^ Few prospective studies have assessed GI‐cancer risks in relation to more direct or precise measurements of body fat [captured via bioimpedance, dual‐energy X‐ray absorptiometry (DEXA) or magnetic resonance imaging (MRI)],^9^, ^10^, ^11^, ^12^, ^13^, ^14^, ^15^, ^16^ and only two studies (with at least 300 cases) have investigated fat‐free mass, with mixed findings.^9^, ^14^

Importantly, there is limited evidence from prospective studies in China where the mean BMI is much lower than that of many Western countries^17^, ^18^; studies in China could therefore provide more detailed insights into GI‐cancer risks at the lower BMI spectrum. Moreover, at a given BMI, Chinese adults were found to have a higher body fat percentage (BF%) and a greater propensity to abdominal obesity compared to Caucasians, and so evidence from Western cohorts may not be generalisable to the Chinese population.^17^, ^19^ Previous prospective studies on adiposity and GI‐cancers in China had several limitations, such as the lack of more precise measures of body fat,^20^, ^21^, ^22^, ^23^ the use of adiposity measurements from a single snapshot only (which do not reflect usual adiposity levels),^13^, ^20^, ^24^, ^25^ or crude adjustment for confounders.^20^, ^25^ Furthermore, only two studies have examined young adulthood adiposity (with CRC only)^22^, ^24^ or the independent role of general versus central adiposity,^23^, ^24^ and none investigated fat‐free mass or associations by SC subsites (cardia vs. non‐cardia).

To address these knowledge gaps, we conducted a detailed investigation of the associations of general (BMI, BF%, fat mass), central (WC and WHR) and young adulthood (BMI at age 25) adiposity and fat‐free mass with the risks of EC, SC and CRC, in the prospective China Kadoorie Biobank. We also assessed whether central adiposity had an independent association* with risks beyond that of general adiposity, and vice versa, and attempted to disentangle the associations of fat‐free and fat mass.

## 2 MATERIALS AND METHODS

The China Kadoorie Biobank (CKB) study design and ethics approval have been described in detail previously.^26^, ^27^ In brief, CKB is a prospective cohort study involving 512,715 adults (aged 30–79 years at baseline) recruited during 2004–2008 from five urban and five rural areas in China, including a high‐risk area for EC (Huixian, in Henan province). The baseline assessment included an interviewer‐administered electronic questionnaire (covering socio‐demographics, diet, lifestyle factors and medical and reproductive history), physical measurements and blood‐sample collection. Two resurveys were conducted in late 2008 and 2013–2014, respectively, amongst 5% of surviving participants randomly‐selected from the original cohort.

### 2.1 Assessment of body size and composition

Measurements of body size and composition were carried out by trained health workers using standardised procedures, and typically taken with participants wearing light‐clothing and no shoes and recorded to the nearest 0.1 cm or 0.1 kg. Height was measured using a portable stadiometer. Weight was measured with the TANITA‐TBF‐300GS body composition analyser, with the weight of clothing (0.5 kg for summer; 1.0 kg for spring/autumn; 2.0–2.5 kg for winter) subtracted. BMI was derived by dividing weight (kg) by the square of standing height (m^2^). BF% was estimated by the TANITA device using bio‐electrical impedance. Fat mass was derived by multiplying body weight and BF%, and fat‐free mass derived by subtracting fat mass from body weight. WC was measured halfway between the lowest rib and the iliac crest, while hip circumference (HC) was measured around the maximum circumference of the buttocks. WC and HC were usually measured over undergarments only. WHR was calculated by dividing WC by HC. Participants self‐reported their weight at age 25 and data were recorded as missing if unknown; BMI25 was derived using this variable and the measured baseline height.

### 2.2 Follow‐up and outcome ascertainment

Participants were followed up via record linkage, using their unique national identification number, with local death and disease registries and national health insurance databases (covering >96% of CKB participants), supplemented by active follow‐up each year. All events were coded using the International Classification of Diseases, 10th Edition (ICD‐10), by trained staff. Event adjudication (i.e. review of medical notes to confirm diagnoses and to retrieve additional information such as cancer histopathology subtype) was completed for a subset of cancer events.

Our main study outcomes were incident EC (ICD‐10: C15), SC (C16) and CRC (C18–C20); event adjudication data showed that 87% of EC cases reviewed were ESCC. In addition, we explored associations with subsites of SC and CRC, including cardia (C16.0) and non‐cardia (C16.1–C16.9) SC, and colon (C18) and rectal (C19–C20) cancer. Participants contributed person‐years from their recruitment date until the occurrence of the outcome of interest, death (from any cause), loss to follow‐up or the study censoring date (31 December 2016), whichever came first.

### 2.3 Statistical analysis

The categorisation of each adiposity trait is documented in Table S1. Individuals with a self‐reported history of cancer at baseline (*n* = 2578) and/or missing or extreme baseline adiposity measurements (*n* = 489) were excluded. Participants with missing data on BMI25 (*n* = 81,816 after the above exclusions) were excluded from the analyses of BMI25. Prevalence or means of baseline characteristics were calculated across adiposity categories, directly standardised to the age, sex and study area structure of the CKB study population, where appropriate. Relationships between different adiposity traits were assessed using Pearson partial correlation coefficients.

Cox regression models were used to obtain hazard ratios (HRs) and 95% confidence intervals (95% CIs) for the associations of adiposity measures, in categories and as continuous traits, with risks of incident GI‐cancers. Cox models were stratified by age‐at‐risk (10‐year bands) and sex, and adjusted for study area, education, household‐income, family history of cancer, smoking, alcohol consumption, physical activity (in MET‐hours/day) and key dietary factors for each cancer. For categorical exposures with more than two levels, 95% CIs were calculated using the floating absolute risk method.^28^ To obtain the HR per ‘unit’ (e.g. per SD or 5 kg/m^2^) increase in ‘usual’ adiposity, the log‐HR and SE associated with each ‘unit’ change in baseline adiposity measure were divided by the corresponding regression dilution ratio (RDR, estimated from the baseline and second resurvey measurements using the MacMahon‐Peto method,^29^, ^30^ Table S2).

We performed subgroup analyses by sex, urban versus rural areas, ever‐regular versus never‐regular smokers and/or alcohol‐drinkers, and for EC, by high‐risk (Huixian) versus non‐high‐risk areas. To minimise potential reverse causation bias, we conducted sensitivity analyses excluding the first 3 (or 5) years of follow‐up, plus individuals with any self‐reported prior chronic diseases and/or poor self‐rated health at baseline. Other sensitivity analyses included: (1) using ESCC cases confirmed via event adjudication as outcome (to assess consistency with the main EC endpoint); (2) separate analyses by subsites of SC and CRC (to test for heterogeneity) and (3) stratification by 5‐year age‐at‐risk, sex and 10 study areas (to better account for confounding by age and region).

To assess the independent associations and relative importance of general versus central adiposity, further analyses were conducted with general adiposity traits (i.e. BMI, BF%, fat mass) adjusted for WC, and central adiposity traits (i.e. WC, WHR) adjusted for BMI. We also conducted analyses of fat mass adjusting for fat‐free mass and height (and vice versa for fat‐free mass). To minimise potential issues of multicollinearity when mutually‐adjusting for highly‐correlated adiposity traits, the 'simple' residuals method was also used for comparison with conventional adjustment (Supplementary Method).^31^, ^32^, ^33^

## 3 RESULTS

### 3.1 Patterns and correlates of adiposity traits

Amongst the 509,648 study participants, the mean (SD) baseline BMI was 23.7 (3.4) kg/m^2^ and 33% were overweight or obese (i.e. BMI ≥ 25 kg/m^2^) at baseline (Table 1). Considerable regional differences in adiposity levels were observed, with most measures higher in urban compared with rural areas (Figure S1). Individuals with higher levels of adiposity (BMI, BF%, WC) were more likely to have higher household income, be ever‐regular alcohol‐drinkers, have lower physical activity levels and have had prior chronic diseases, and less likely to be ever‐regular smokers (Table 1 and Tables S3 and S4).

**TABLE 1:** Baseline characteristics of participants by sex and BMI categories.^a^

	Men	Women	All participants	BMI (kg/m^2^, WHO international cut‐offs)			
				<18.5	18.5–24.9	25.0–29.9	≥30.0
Number (%) of participants	209,062 (41.0)	300,586 (59.0)	509,648 (100.0)	22,044 (4.3)	319,756 (62.7)	147,179 (28.9)	20,669 (4.1)
Socio‐demographic factors							
Age, year	52.8 ± 10.9	51.4 ± 10.5	52.0 ± 10.7	55.0 ± 12.1	51.5 ± 10.8	52.1 ± 10.2	52.1 ± 10.4
Female, %	NA	NA	59.0	59.7	57.7	60.7	71.1
Urban, %	43.4	44.8	44.1	31.8	40.1	52.9	58.8
High school education or above, %	26.1	17.5	21.0	20.1	21.2	21.3	19.5
Household income ≥ 20,000 yuan/year, %	45.6	40.8	42.7	36.9	42.4	44.7	44.7
Lifestyle and dietary factors^b^							
Ever‐regular smokers, %	74.2	3.3	32.4	35.8	32.9	30.8	31.0
Ever‐regular alcohol‐drinkers, %	41.9	3.0	19.0	16.7	18.8	19.5	19.8
Total physical activity, MET‐hr/day	22.4 ± 15.3	20.2 ± 12.8	21.1 ± 13.9	20.8 ± 13.4	21.6 ± 14.1	20.4 ± 13.4	19.0 ± 12.3
Regular intake of fruits, %	23.4	31.3	28.2	23.2	27.7	29.7	30.1
Regular intake of meat, %	52.7	43.4	47.2	42.7	46.7	49.2	49.8
Has family history of cancer, %	17.1	16.9	17.0	15.1	16.8	17.7	17.4
Body composition							
Height, cm	165.3 ± 6.5	154.1 ± 6.0	158.7 ± 8.3	158.7 ± 8.5	158.6 ± 8.1	158.9 ± 8.4	159.0 ± 8.5
Weight, kg	64.3 ± 10.9	56.6 ± 9.4	59.8 ± 10.7	44.3 ± 5.3	55.9 ± 7.2	67.8 ± 8.0	80.2 ± 9.4
Body mass index, kg/m^2^	23.5 ± 3.2	23.8 ± 3.4	23.7 ± 3.4	17.6 ± 0.8	22.1 ± 1.7	26.8 ± 1.3	31.7 ± 1.7
Body fat percentage, %	22.1 ± 6.2	32.1 ± 7.1	27.9 ± 8.4	16.6 ± 4.3	25.4 ± 6.4	33.5 ± 6.8	40.0 ± 7.9
High BF% [>25 (M), >35 (F)], %	30.8	32.3	31.5	0.1	10.9	71.4	96.6
Fat mass, kg	14.7 ± 6.2	18.7 ± 6.9	17.0 ± 6.9	7.3 ± 1.8	14.2 ± 4.0	22.5 ± 4.5	31.8 ± 6.3
Fat‐free mass, kg	49.6 ± 6.1	37.9 ± 4.1	42.7 ± 7.6	37.1 ± 5.4	41.7 ± 6.7	45.3 ± 8.3	48.4 ± 10.0
Waist circumference, cm	82.1 ± 9.8	79.1 ± 9.5	80.3 ± 9.7	65.7 ± 5.0	76.6 ± 6.7	88.1 ± 6.6	98.9 ± 7.3
Abdominal obesity [>94 (M), >80 (F)], %^c^	11.6	43.5	30.2	0.4	14.0	61.2	96.2
Hip circumference, cm	90.7 ± 6.8	91.2 ± 6.9	90.9 ± 6.8	81.8 ± 4.2	88.6 ± 4.9	95.6 ± 5.0	103.3 ± 6.1
Waist‐to‐hip ratio	0.90 ± 0.06	0.87 ± 0.07	0.88 ± 0.07	0.80 ± 0.06	0.86 ± 0.06	0.92 ± 0.06	0.96 ± 0.07
Body mass index at age 25, kg/m^2^ ^d^	21.9 ± 2.4	21.9 ± 2.7	21.9 ± 2.6	20.0 ± 2.4	21.5 ± 2.4	22.6 ± 2.6	23.9 ± 3.1

Abbreviations: BF%, body fat percentage; BMI, body mass index; F, female; M, male; MET‐hr/day, metabolic equivalents of task hours per day; WHO, World Health Organization.

^a^ Plus–minus values are means ± SD; Data were directly standardised to the age, sex and region structure of the study population when appropriate.

^b^ Ever‐regular smokers include current‐ and ex‐regular smokers; ever‐regular alcohol‐drinkers include current‐regular, ex‐regular and reduced‐intake drinkers; regular dietary intake refers to consumption on ≥4 days/week.

^c^ With reference to the World Health Organization's cut‐off points for increased risk of metabolic complications.^67^

^d^ Body mass index at age 25 was only available in 427,832 participants.

BMI was highly correlated with BF%, fat mass and WC (*r* > .8), but only moderately correlated with fat‐free mass (.54) and WHR (.56; Table S5). Fat mass and fat‐free mass were moderately correlated (.45), while BMI25 was only weakly correlated with baseline BMI (.31).

### 3.2 Associations of adiposity with GI‐cancer risks

**FIGURE 1:**
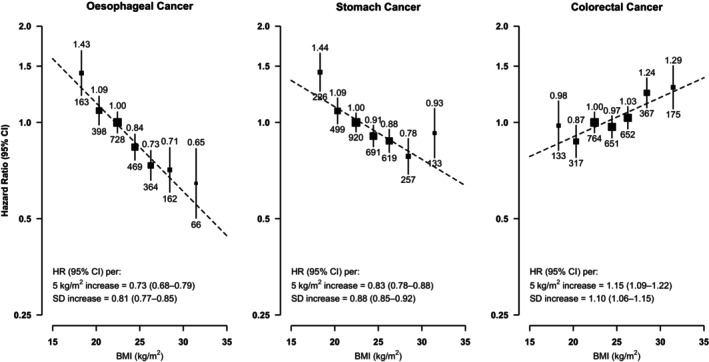
Adjusted hazard ratios (HRs) for GI‐cancers by usual levels of BMI. Analyses were stratified by age‐at‐risk (10‐year bands) and sex, and adjusted for 10 study areas, education level, household income level, family history of cancer, smoking (four categories), alcohol consumption (six categories), physical activity and dietary factors that were significantly associated with the cancer of interest (i.e. fruit, soy, preserved vegetables and spicy food intake for oesophageal cancer; and fruit intake for stomach cancer). HRs were plotted against the mean BMI values at second resurvey in baseline‐defined BMI categories. Vertical lines represent floated 95% CIs. The size of each square is inversely proportional to the variance of the log‐HR. Numbers above the squares are HRs and numbers below are number of events. Sex‐specific SDs were used. BMI, body mass index; CI, confidence interval; SD, standard deviation.

During 5.1 million person‐years of follow‐up (median = 10.1 years), 2350, 3345 and 3059 incident cases of oesophageal, stomach and colorectal cancers were recorded, respectively. For BMI, there was an inverse log‐linear association with EC throughout the BMI range studied, with each SD increase in usual BMI associated with a 19% (95% CI 15–23%) lower risk [HR per 5 kg/m^2^ increase = 0.73 (0.68–0.79); Figure 1 and Figure S2]. There was also an inverse association of BMI with SC which was approximately log‐linear up to ~28 kg/m^2^, and a positive association with CRC which was approximately log‐linear above ~18.5 kg/m^2^. Compared to those with ‘normal’ BMI as defined by the WHO international criteria (see Table S1), the adjusted HRs for ‘overweight’ and ‘obese’ individuals were 0.76 (95% CI 0.69–0.84) and 0.68 (0.53–0.87) for EC, 0.86 (0.80–0.94) and 0.95 (0.79–1.14) for SC, and 1.14 (1.05–1.23) and 1.33 (1.13–1.56) for CRC, respectively (Figure S2).

**FIGURE 2:**
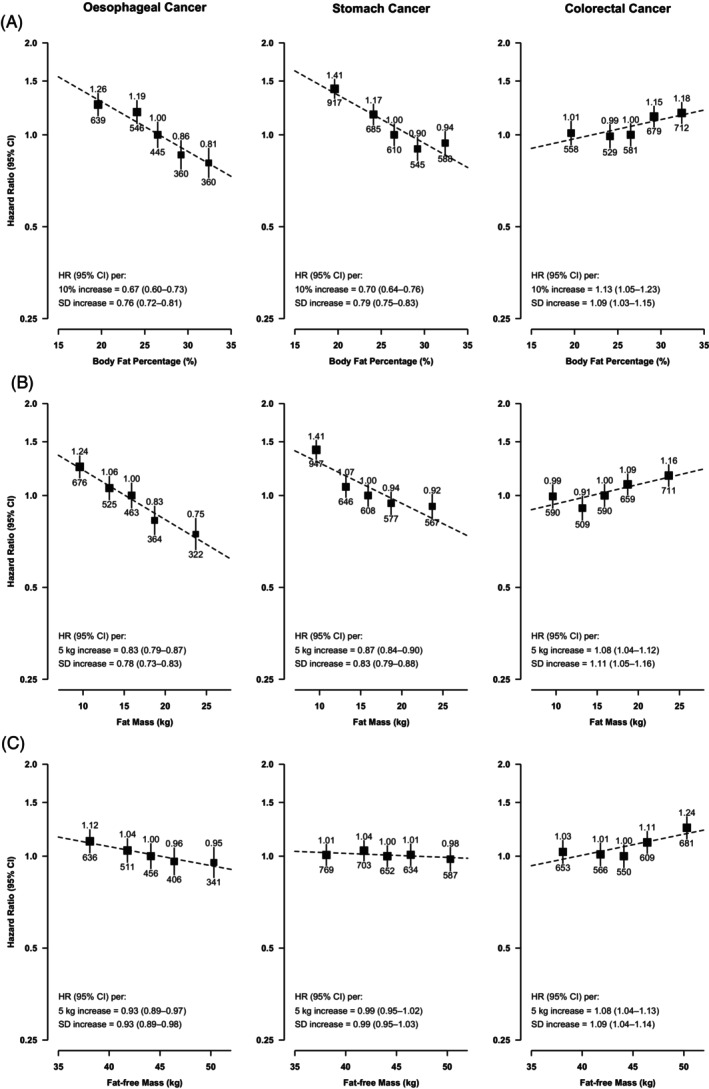
Adjusted hazard ratios (HRs) for GI‐cancers by usual levels of: (A) body fat percentage, (B) fat mass and (C) fat‐free mass. Conventions as per Figure 1.

BF% and fat mass were also inversely and log‐linearly associated with the risks of EC [0.76 (0.72–0.81) and 0.78 (0.73–0.83) per SD higher usual BF% and fat mass, respectively] and SC [0.79 (0.75–0.83) and 0.83 (0.79–0.88) per SD higher BF% and fat mass], although as with BMI the inverse association between BF% and SC appeared to be attenuated for the highest BF% quintile (Figure 2). For fat‐free mass, a weak inverse log‐linear association with EC was observed [0.93 (0.89–0.98) per SD higher usual fat‐free mass], but there was no association with SC. For CRC, there were positive associations with BF%, fat mass and fat‐free mass, with the associations seemingly driven by the two highest quintiles of each trait (Figure 2). The associations of fat and fat‐free mass with the three cancers remained similar after additional adjustment for height and mutual adjustment for each other (Figure S3).

**FIGURE 3:**
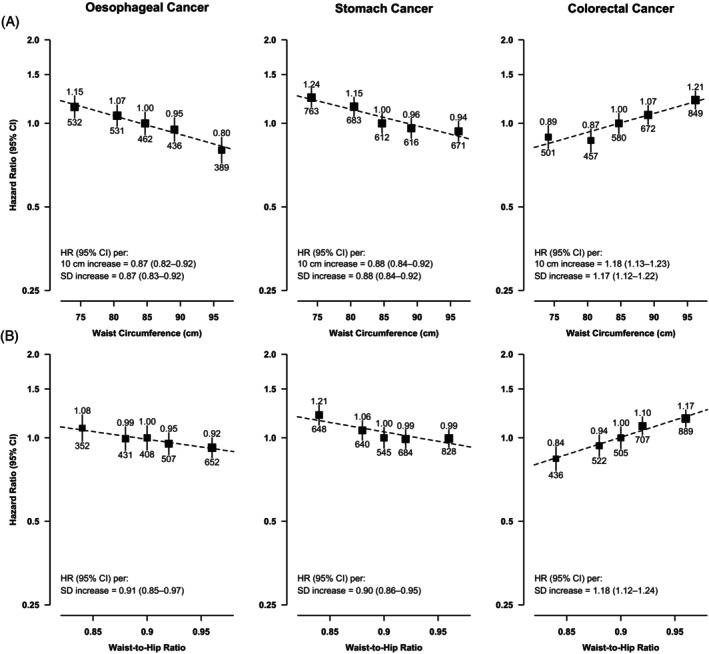
Adjusted hazard ratios (HRs) for GI‐cancers by usual levels of: (A) waist circumference and (B) waist‐to‐hip ratio. Conventions as per Figure 1.

As for central adiposity, WC and WHR were inversely associated with the risks of EC and SC and positively associated with CRC, in an approximately log‐linear manner (Figure 3). Each SD increase in usual WC was associated with a 13% (8%–17%) and a 12% (8%–16%) lower risk of EC and SC, respectively, and a 17% (12%–22%) higher risk of CRC. Across the different adiposity traits, general adiposity traits appeared to be slightly more strongly associated with EC risk compared to central adiposity traits, while BF% appeared to be most strongly associated with SC risk (Figure S4). For CRC, the strength of association was similar across all adiposity traits examined.

**FIGURE 4:**
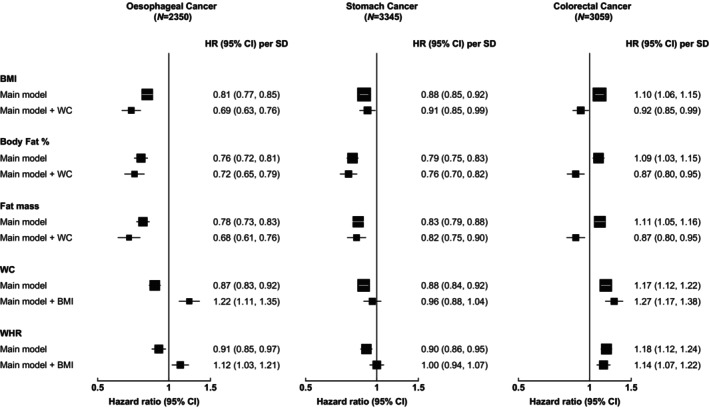
Associations between usual general and central adiposity levels and GI‐cancer risks, with mutual adjustments. In the main model, analyses were stratified by age‐at‐risk (10‐year bands) and sex, and adjusted for 10 study areas, education level, household income level, family history of cancer, smoking, alcohol consumption, physical activity and dietary factors (fruit, soy, preserved vegetables and spicy food intake for oesophageal cancer; and fruit intake for stomach cancer). The size of each square is inversely proportional to the variance of the log‐HR. Sex‐specific SDs were used. BMI, body mass index; CI, confidence interval; HR, hazard ratio; SD, standard deviation; WC, waist circumference; WHR, waist‐to‐hip ratio.

After adjusting for central adiposity (WC), BMI, BF% and fat mass remained inversely associated with EC and SC, while the positive associations with CRC became inverse (Figure 4). On the other hand, after adjusting for general adiposity (BMI), the inverse associations of central adiposity with EC became positive and the inverse associations with SC were attenuated to the null, while the positive associations with CRC remained unchanged. Mutual adjustment using the residuals method gave very similar conclusions to the conventional adjustment (Figure S5).

Young adulthood adiposity (BMI25) was positively associated with EC risk [1.16 (1.03–1.31) per usual 5 kg/m^2^ increase], and with SC and CRC risks, with the associations approximately log‐linear up to ~25 kg/m^2^ for SC and up to ~23–24 kg/m^2^ for CRC (Figure S6). Associations were directionally‐consistent after additional adjustment for baseline BMI (Figure S6).

### 3.3 Subgroup and sensitivity analyses

In analyses using confirmed ESCC (726 cases), the associations with BMI, BF% and fat mass were consistent with those observed for overall EC, but the associations with other adiposity traits were no longer statistically‐significant (Table S6). Heterogeneity (in associations) between SC subsites was found, with BMI, BF%, fat mass and WC inversely associated with non‐cardia SC (2957 cases) but not with cardia SC (388 cases, p‐heterogeneity < 0.05, Table S6), and the shape and magnitude of associations with non‐cardia SC were very similar to those found for overall SC (Figure S7). Associations did not appear to differ for colon and rectal cancer (Table S6).

The inverse associations of BMI, fat mass and WC with EC were stronger in ever‐regular, than in never‐regular, smokers (p‐heterogeneity < 0.05; Figures S8–S13). WC and WHR were more strongly associated with CRC in ever‐regular, compared to never‐regular, alcohol‐drinkers, and in men compared to women (p‐heterogeneity < 0.05, Figures S12 and S13). Fat‐free mass was inversely associated with EC risk in the non‐high‐risk areas but not in the high‐risk rural area (Huixian; p‐heterogeneity = 0.01; Table S7). Other adiposity — GI‐cancer associations did not differ significantly by smoking, alcohol‐consumption, sex, urban–rural areas or by high‐risk versus non‐high‐risk areas for EC.

The main results remained largely consistent in the sensitivity analyses stratified by 5‐year age‐at‐risk, sex and 10 study areas (Table S8). After exclusions of the first 3 years of follow‐up and participants with any prior chronic diseases and/or poor self‐rated health, the inverse associations of most adiposity traits with EC and SC were attenuated (central adiposity and fat‐free mass were no longer significant, although estimates for fat‐free mass were largely unchanged). Associations with CRC remained similar (Table S9).

## 4 DISCUSSION

Our study showed that general adiposity (as measured by BMI, BF% and fat mass) was inversely associated with EC and SC risks, independently of central adiposity. Associations between central adiposity and these two cancers were less clear; although there was some evidence of inverse associations, these were not consistently observed in our sensitivity analyses or after accounting for general adiposity. Associations appeared to differ by SC subsites, with inverse associations with non‐cardia SC but no significant associations with cardia SC (but case numbers were low for cardia). For CRC, all adiposity traits examined were positively associated with risk, but the associations with central adiposity seemed to be more robust and were independent of general adiposity. We also found fat‐free mass to be potentially inversely associated with EC and positively associated with CRC, independently of fat mass and height.

### 4.1 General and central adiposity with GI‐cancers

#### 4.1.1 Oesophageal cancer

The inverse association between BMI and EC (primarily ESCC) found in our study is consistent with the findings from most previous ESCC studies,^13^, ^20^, ^33^ including the latest WCRF dose–response meta‐analysis of eight prospective studies with 4348 cases [summary HR = 0.64 (0.56–0.73) per 5 kg/m^2^ increase].^6^ As BMI does not differentiate between fat and fat‐free mass, the inverse association with BMI may be due to higher ESCC risk associated with lower fat‐free (muscle and bone) mass resulting from poorer nutrition, particularly since malnutrition and/or micronutrients deficiency have been suggested to be potential risk factors of ESCC.^20^, ^34^, ^35^ Our study is the first prospective cohort to investigate the relationship between fat‐free mass and ESCC risk, and higher fat‐free mass was indeed associated with lower risk (independent of fat mass and height) in our main analysis. This association, however, was no longer statistically‐significant when restricted to confirmed ESCC or never‐regular smokers, or after various exclusions, which could be due to the much‐reduced statistical power, or it may suggest that the inverse association in the main analysis was due to residual confounding from smoking and/or reverse causation, and further investigation is needed.

Interestingly, we also found higher BF% and fat mass to be associated with lower EC (ESCC) risk, consistent with findings from UKB, the only other prospective investigation of BF% and ESCC identified [HR = 0.39 (0.21–0.72) for highest (>35.3%) vs. lowest (<26.9%) BF% tertile, p‐trend = 0.01, 124 cases].^12^ We additionally showed that risk decreased with no apparent BF% threshold. As there are no compelling biological mechanisms supporting a protective role of body fat in relation to ESCC, these associations could be due to reverse causation and/or residual confounding. However, our inverse associations remained after excluding the first 3 (or 5) years of follow‐up and participants with prior chronic diseases or poor self‐rated health, in‐line with findings from other prospective studies (mostly on BMI) that attempted to minimise reverse causation, including the Million Women Study (MWS) that observed an inverse association with BMI even after excluding 15 years of follow‐up.^12^, ^33^, ^36^, ^37^, ^38^ In addition, our study, MWS and the WCRF meta‐analysis all found general adiposity to be inversely associated with ESCC in either never, never‐regular or non‐smokers,^33^, ^39^ suggesting that the observed inverse relationships are not likely to be fully explained by residual confounding from smoking.

Previous prospective studies on central adiposity and ESCC risk have reported mixed findings,^12^, ^33^, ^36^, ^38^, ^40^, ^41^ although the largest studies among Europeans (MWS) and Asians [the Korean National Health Insurance Corporation (NHIC) study] both found inverse associations.^33^, ^41^ We similarly observed inverse associations with central adiposity in our main analyses, but they were attenuated to the null after various exclusions, suggesting that they may be explained (at least partly) by reverse causation. Our study also showed that the inverse associations with central adiposity turned positive after adjusting for general adiposity, while general adiposity remained independently and inversely associated with risk. This aligns with the results from EPIC and the Korean‐NHIC, but differs from the MWS findings where central adiposity remained inversely associated with ESCC after accounting for general adiposity.^33^, ^36^, ^41^ Overall, the current evidence for ESCC seems to suggest potential benefits from general adiposity, but the role of abdominal adiposity is less clear.

#### 4.1.2 Stomach cancer

Similar to our findings, the WCRF meta‐analysis (28,916 cases from 19 studies), the Asia Cohort Consortium (ACC; 8997 cases from 13 studies) and the UK CPRD study (3337 cases) also found an inverse association between BMI and overall SC up to ~23–25 kg/m^2^, with some indication of increased SC risk at the higher end of BMI.^42^, ^43^, ^44^ Several previous prospective studies have investigated the associations of BF% or fat mass with SC risk,^9^, ^11^, ^12^, ^25^, ^45^ but besides CKB, only UKB had >300 SC cases and adequate adjustment for key confounders.^11^ Contrary to the inverse association in CKB, a significant positive association between BF% and overall SC was found in UKB [HR = 1.16 (1.08–1.26) per SD increase, 747 cases].^11^ Similarly, while central adiposity measures were inversely associated with overall SC risk in our study, most previous prospective studies have either reported positive or null associations.^11^, ^46^, ^47^

One potential explanation for these differences may be related to the higher proportion of non‐cardia SC in CKB (2957 out of 3345 SC cases) and cardia SC in other cohorts (e.g. 404 out of 747 SC cases in UKB).^11^ Existing epidemiological and mechanistic evidence seems to suggest that the association with adiposity differs by SC subsites, with higher adiposity associated with higher cardia SC risk (possibly due to gastro‐oesophageal reflux) but the association with non‐cardia SC remains unclear since few studies had adequate power.^7^, ^20^, ^38^, ^42^, ^44^, ^48^ The ACC and the *Helicobacter pylori* Biomarker Cohort Consortium both observed a reverse‐J‐shaped (or U‐shaped) association between BMI and non‐cardia SC risk (6314 and 1591 cases, respectively), but other adiposity traits were not examined.^44^, ^49^ As the largest prospective study on non‐cardia SC that assessed multiple bioimpedance and anthropometric traits, we provided new evidence that most adiposity traits were inversely associated with risk, with some indication of a reverse‐J‐shaped relationship for BMI and BF%, although further large‐scale studies are needed to confirm the robustness and shape of these associations.

#### 4.1.3 Colorectal cancer

The positive association of BMI with CRC risk observed in our study is supported by a large body of existing literature.^8^, ^50^, ^51^ However, besides CKB, only seven previous prospective investigations (three conducted in UKB) have examined the associations of BF% or fat mass with CRC.^9^, ^10^, ^11^, ^14^, ^15^, ^16^, ^52^ Consistent with our findings, UKB, the Nurses' Health Study (NHS) and the Health Professionals Follow‐up Study (HPFS) also reported a significant positive association between BF% and CRC risk, with a similar magnitude of association to BMI [e.g. HR = 1.09 (1.05–1.13) per SD increase in BF% in UKB, with 4394 cases].^11^, ^14^ As for fat‐free mass, only two previous prospective studies with >1000 cases were identified.^9^, ^14^ In the NHS and HPFS, fat‐free mass was positively associated with CRC risk [HR = 1.12 (1.06–1.19) in women and 1.18 (1.10–1.26) in men per SD increase], but no significant association with colon cancer was found in an UKB investigation that additionally‐adjusted for fat mass.^9^, ^14^ In CKB, where fat‐free mass is on average lower than those reported in Western populations, fat‐free mass remained positively associated with CRC risk even after accounting for fat mass and height, but more evidence from diverse populations is required to understand and verify this association. The mechanisms underlying this (potential) positive association are unknown, but some speculated that it may be related to insulin‐like growth factor 1 (which has been associated with both higher fat‐free mass and increased CRC risk), or elevated basal metabolic rate in those with higher fat‐free mass (which is related to increased oxidative stress and expression of inflammatory cytokines).^9^, ^53^

Our positive associations between central adiposity measures and CRC are generally in‐line with results from previous meta‐analyses, with the WCRF reporting an HR of 1.02 (1.01–1.03) per 10 cm increase in WC.^8^, ^50^ In particular, our study showed that central adiposity was positively associated with CRC risk independent of general adiposity, but it is unclear whether general adiposity has an independent association. Other prospective studies that assessed the associations of general and central adiposity with mutual adjustment for each other have reported inconsistent results, with the Women's Health Initiative (1908 cases) and the Shanghai Men's Health Study (313 cases) suggesting that central adiposity has an independent, and stronger, association with CRC risk compared to BMI,^22^, ^24^, ^54^ but a pooled analysis of seven European cohorts (591 cases) reported the converse.^50^ Results from other cohorts appeared to differ by sex and/or CRC subsites.^55^, ^56^, ^57^

### 4.2 Young adulthood adiposity (BMI25)

In this study, EC risk was inversely associated with mid/late‐adulthood BMI but positively associated with BMI25, which is consistent with findings from the Japan Public Health Center‐based (JPHC) Prospective Study.^37^ We additionally showed that the positive association with BMI25 was independent of mid/late‐adulthood BMI. As for SC, there was some evidence of positive associations of BMI25 with overall and non‐cardia SC in CKB, and with cardia SC in the NIH‐AARP and PLCO cohorts,^58^ but no significant association in the Netherlands Cohort Study possibly due to limited power.^59^ For EC and SC, differences in the direction of association between mid/late‐ and early‐adulthood BMI may be related to the different extent of biases in these two traits. For example, the associations with early‐adulthood BMI are less affected by reverse causation compared to mid/late‐adulthood BMI, but early‐adulthood BMI is often self‐reported and thus subject to recall error/bias. Alternatively, BMI at different time‐points across the lifespan may have different impacts on cancer risk in later life, but further evidence from studies following individuals from early‐adulthood to late‐adulthood is needed. As for CRC, our observation of a positive association with BMI25 is generally consistent with findings from other Western cohorts.^60^, ^61^, ^62^

### 4.3 Strengths and limitations

Apart from being one of the largest prospective studies on adiposity in relation to EC and SC risks (particularly ESCC and non‐cardia SC), the strengths of our study include the investigation of total BF%, fat mass and fat‐free mass in addition to traditional anthropometric measures. In particular, this study is one of the few to assess and compare the independent associations of fat and fat‐free mass with GI‐cancer risks, which may provide some insight into the aforementioned malnutrition hypothesis relating to BMI and ESCC. Moreover, body size and composition were measured by trained staff in CKB, which are less prone to differential misclassification than self‐reported measures.^63^ Using repeat measurements, we were able to correct for regression dilution bias and the resulting HRs may better approximate risks associated with 'usual' adiposity levels.

One limitation of our study is potential residual confounding from factors that are less accurately measured (e.g. diet) or unmeasured (e.g. *H. pylori* infection, data for which is currently only available in 0.8% of the cohort). Our preliminary analyses in the CKB sub‐cohort with infection data (*n* = 2000), however, showed that adiposity levels were not significantly associated with *H. pylori* infection status (*p* > .05 for all traits). Another limitation is the inability to exclude more than 5 years of follow‐up (to avoid losing too many cases), and given the long latency of cancer, reverse causation could not be eliminated as an explanation for the inverse associations with EC and SC. Thirdly, histological subtype information was obtained from preliminary data from event adjudication, and this was only available in a subset of EC cases. Findings from analyses using confirmed ESCC cases should therefore be interpreted with care, particularly because of the much‐reduced statistical‐power compared to the main EC analyses. Furthermore, CKB only has bioimpedance estimations of total BF%, but not direct measurements of subcutaneous, visceral and ectopic fat. Although bioimpedance is not the gold standard for measuring body composition and its accuracy could be influenced by participants' hydration status and the proprietary equations used, this technique has been validated against hydrodensitometry and studies have shown high correlations between bioimpedance estimations (from TANITA devices) and DEXA measurements for total BF%, fat and fat‐free mass.^64^, ^65^, ^66^ Finally, self‐reported weight at age 25 is subject to recall bias and there was a considerable amount of missing data (~16% of participants), but multiple imputation was not adopted as it relies on other assumptions.

## 5 CONCLUSIONS

Overall, in this large and relatively lean Chinese cohort, higher general adiposity was associated with lower risks of EC and SC, with and without accounting for central adiposity. There was also some evidence of inverse associations between central adiposity and EC and SC, but these associations were less robust. For CRC, all adiposity traits examined were positively associated with risk, but the associations with central adiposity seemed to be more robust. Besides body fat, our study also provided new evidence for fat‐free mass, with a suggestive inverse association with EC and a positive association with CRC. Further research in diverse populations with different patterns of adiposity and GI‐cancer rates and subtypes are needed to confirm our observed associations.

## AUTHOR CONTRIBUTIONS

**Wing Ching Chan:** Conceptualization; formal analysis; investigation; methodology; writing – original draft; writing – review and editing. **Iona Millwood:** Conceptualization; supervision; writing – review and editing. **Christiana Kartsonaki:** Methodology; writing – review and editing. **Huaidong Du:** Writing – review and editing. **Daniel Schmidt:** Data curation; resources; software. **Rebecca Stevens:** Data curation; resources; software. **Junshi Chen:** Project administration; resources. **Pei Pei:** Project administration; resources. **Canqing Yu:** Project administration; resources. **Dianjianyi Sun:** Project administration; resources. **Jun Lv:** Project administration; resources. **Xianyong Han:** Project administration; resources. **Liming Li:** Funding acquisition; resources. **Zhengming Chen:** Funding acquisition; supervision; writing – review and editing. **Ling Yang:** Conceptualization; supervision; writing – review and editing.

## FUNDING INFORMATION

The CKB baseline survey and the first re‐survey were supported by the Kadoorie Charitable Foundation in Hong Kong. The long‐term follow‐up has been supported by Wellcome grants to Oxford University (212946/Z/18/Z, 202922/Z/16/Z, 104085/Z/14/Z, 088158/Z/09/Z) and grants from the National Natural Science Foundation of China (8223000863, 82192900, 82192901, 82192904) and from the National Key Research and Development Program of China (2016YFC0900500).The UK Medical Research Council (MC_UU_00017/1,MC_UU_12026/2, MC_U137686851), Cancer Research UK (C16077/A29186; C500/A16896) and the British Heart Foundation (CH/1996001/9454), provide core funding to the Clinical Trial Service Unit and Epidemiological Studies Unit at Oxford University for the project.

## CONFLICT OF INTEREST STATEMENT

The authors declare no conflicts of interest.

## ETHICS STATEMENT

The China Kadoorie Biobank (CKB) complies with all the required ethical standards for medical research on human subjects. Ethical approvals were granted and have been maintained by the relevant institutional ethical research committees in the UK and China, as listed below. Informed consent was obtained from all participants included in the study. UK ethical approvals: Kadoorie Study of Chronic disease in China (KSCDC) – Baseline, Oxford Tropical Research Ethics Committee (OxTREC) (2005) OxTREC Ref: 025‐04; China Kadoorie Biobank (A prospective study of environmental and genetic causes of premature death in 500,000 Chinese adults) – Second resurvey (2013) OxTREC Reference: 1024‐13. China ethical approvals: Kadoorie Study of Chronic disease in China (KSCDC) – Baseline, Chinese Centre for Disease Control and Prevention. Ethical Review Committee (2004) Approval Notice 005/2004; Kadoorie Study of Chronic disease in China (KSCDC) – Second Phase, Chinese Academy of Medical sciences/Peking Union Medical College, Ethical Committee (2010) 24th December 2010; Kadoorie Study of Chronic disease in China (KSCDC) – Second Resurvey, Chinese Academy of Medical Sciences/Peking Union Medical College, Ethical Committee (2013) Ref: X1303262001; China Kadoorie Biobank–Third Phase, Peking University, Ethical Committee (2020) Ref: IRB00001052‐20040.

## Supporting information


Data S1

## Data Availability

Access to the China Kadoorie Biobank data may be made available following a formal data request to ckbaccess@ndph.ox.ac.uk, subject to the institution's data access policies. Preliminary event adjudication data are not publicly available. Further information is available from the corresponding author upon request.

## Endnote

* “Independent association” is used in this article to refer to the association of one trait (e.g. central adiposity) with cancer risk after adjusting for another (e.g. general adiposity); we acknowledge that general and central adiposity, as well as fat and fat‐free mass, are unlikely to be “truly independent” of each other in a real‐world setting.
