# Supplementary material for: Adiposity and risks of gastrointestinal cancers: A 10‐year prospective study of 0.5 million Chinese adults
Source: Int J Cancer. 2024 Dec 31;156(11):2094–106. doi: 10.1002/ijc.35303 (PMC11970548; doi:10.1002/ijc.35303)
Supplement: Supplementary file 1 — Data S1 [file IJC-156-2094-s001.pdf]

Supplementary Material

Adiposity and risks of gastrointestinal cancers: a 10-year prospective study of 0.5 million Chinese adults

Wing Ching Chan, Iona Millwood, Christiana Kartsonaki, Huaidong Du, Daniel Schmidt, Rebecca Stevens, Junshi Chen, Pei Pei, Canqing Yu, Dianjianyi Sun, Jun Lv, Xianyong Han, Liming Li, Zhengming Chen, Ling Yang, for the China Kadoorie Biobank (CKB) collaborative group

Table of Contents

Supplementary Method 2

Table S1. Categorisation of adiposity traits 2

Table S2. Regression dilution ratios (RDR) for adiposity traits 3

Table S3. Baseline characteristics of participants by waist circumference quintiles* 4

Table S4. Baseline characteristics of participants by body fat percentage quintiles* 5

Table S5. Pearson partial correlations between baseline body composition measures* 6

Table S6. Adjusted HRs (95% CIs) for GI-cancer subtype/ subsites per SD higher usual levels of adiposity* 7

Table S7. Adjusted HRs (95% CIs) for oesophageal cancer per SD higher usual levels of adiposity, stratified by high-risk and non-high-risk areas* 8

Table S8. Associations of usual adiposity with GI-cancers (stratified by 5-year age-at-risk, sex, 10 study areas) 9

Table S9. Adjusted HRs (95% CIs) for GI-cancers per SD higher usual levels of adiposity, excluding the first 3 or 5 years of follow-up, any prior chronic diseases and self-rated poor health 10

Figure S1. Adjusted means of selected baseline adiposity traits by CKB study areas 11

Figure S2. Adjusted HRs for GI-cancers by usual levels of BMI: A) WHO-international cut-offs, B) WHO-Asian cut-offs, and C) CKB cut-offs 12

Figure S3. Associations of baseline fat and fat-free mass with GI-cancers, with mutual adjustments. 13

Figure S4. Comparison across adiposity traits 14

Figure S5. Associations of baseline general and central adiposity with GI-cancers, with mutual adjustments 15

Figure S6. Adjusted HRs for GI-cancers by usual BMI at age 25 16

Figure S7. Associations of all adiposity traits with risk of non-cardia SC 17

Figure S8. Adjusted HRs for GI-cancers per SD increase in usual levels of BMI, by selected baseline characteristics 18

Figure S9. Adjusted HRs for GI-cancers per SD increase in usual levels of body fat %, by selected baseline characteristics 19

Figure S10. Adjusted HRs for GI-cancers per SD increase in usual levels of fat mass, by selected baseline characteristics 20

Figure S11. Adjusted HRs for GI-cancers per SD increase in usual levels of fat-free mass, by selected baseline characteristics 21

Figure S12. Adjusted HRs for GI-cancers per SD increase in usual levels of waist circumference, by selected baseline characteristics 22

Figure S13. Adjusted HRs for GI-cancers per SD increase in usual levels of WHR, by selected baseline characteristics 23

Members of the China Kadoorie Biobank collaborative group 24

References 25

Supplementary Method

In the "simple" residuals method,[1-3] residuals obtained from a linear regression of central adiposity (WC or WHR) on BMI were entered into the Cox model, to assess the association of central adiposity with cancer risk whilst accounting for the effect of general adiposity (BMI). The same principles were applied when studying the effect of general adiposity whilst adjusting for central adiposity, and in the mutual-adjustment of fat and fat-free mass.

Table S1. Categorisation of adiposity traits

**BMI**	WHO international guidelines: "Underweight" (<18.5), "Normal" (18.5-24.9), "Overweight" (25.0-29.9), "Obese" (≥30.0)<br>WHO Asian-specific guidelines: <18.5, 18.5-22.9, 23.0- <27.5, ≥27.5<br>CKB cut-offs ***** : <18.5, <20.5, <23.0, <25.0, <27.5, <30.0, and ≥30.0
**BMI25**	CKB cut-offs, with the last 3 categories combined to improve power.
**BF%, fat mass, fat-free mass, WC, WHR**	Sex-specific quintiles

Abbreviations: WHO= World Health Organization; BMI= body mass index; BMI25= body mass index at age 25; BF%= body fat percentage; WC= waist circumference; WHR= waist-to-hip ratio.

* To better assess the shape of associations, BMI was also categorised into 7 categories, with similar cut-offs as those used in previous CKB publications,[4] which correspond roughly to a combination of both the WHO international and Asian BMI cut-offs.

Table S2. Regression dilution ratios (RDR) for adiposity traits

Adiposity traits	RDR (MacMahon-Peto) *
Body Mass Index, kg/m²	0.92
Body Fat %	0.69
Fat mass, kg	0.78
Fat-free mass, kg	0.91
Waist Circumference, cm	0.83
Waist-to-hip Ratio	0.64
Body Mass Index at age 25, kg/m²	0.71

* Amongst individuals without prior cancer at baseline and those with complete adiposity information at both baseline and second resurvey.

Table S3. Baseline characteristics of participants by waist circumference quintiles*

	Waist circumference (sex-specific) quintiles					
	**Q1**	**Q2**	**Q3**	**Q4**	**Q5**	**All participants**
Number of participants	100,881	100,134	103,307	102,587	102,739	509,648
Waist circumference (cm, adjusted)	67.7 ± 3.5	74.5 ± 2.0	79.7 ± 2.2	85.1 ± 2.5	93.9 ± 5.4	80.3 ± 9.7
**Socio-demographic factors**						
Age, year	50.8 ± 11.3	50.5 ± 10.6	51.3 ± 10.4	52.4 ± 10.3	53.9 ± 10.4	52.0 ± 10.7
Female, %	59.3	58.0	59.4	59.2	59.2	59.0
Urban, %	32.8	37.7	44.2	49.7	53.4	44.1
High school education or above, %	19.9	21.1	21.7	21.7	20.2	21.0
Household income ≥20,000 yuan/year, %	37.8	41.4	43.6	45.0	45.9	42.7
**Lifestyle factors †**						
Ever-regular smokers, %	33.9	32.7	31.7	31.3	32.2	32.4
Ever-regular alcohol drinkers, %	16.9	18.4	19.0	19.7	20.5	19.0
Total physical activity, MET-hr/day	22.1 ± 14.3	21.8 ± 14.2	21.3 ± 14.0	20.6 ± 13.4	19.7 ± 12.7	21.1 ± 13.9
**Regular dietary intake, % ‡**						
Fresh fruits	25.6	27.6	28.7	29.2	29.1	28.2
Fresh vegetables	98.2	98.3	98.3	98.4	98.3	98.3
Meat	44.1	46.4	47.8	48.8	49.5	47.2
**Medical history, %**						
Has family history of cancer	15.8	16.7	16.9	17.8	17.7	17.0
Had any prior chronic diseases §	21.9	20.3	21.0	22.1	25.4	22.4

Abbreviations: MET-hr/day=metabolic equivalents of task hours per day.

* Plus–minus values are means ± SD.

Data were directly standardised to the age, sex, and region structure of the study population when appropriate.

† Ever-regular smokers include current- and ex-regular smokers; ever-regular alcohol drinkers include current-regular, ex-regular, and reduced-intake drinkers.

‡ Regular dietary intake refers to consumption on ≥4 days/week.

§ Chronic diseases included diabetes, chronic heart diseases, stroke/transient ischemic attack, chronic hepatitis/liver cirrhosis, kidney diseases, peptic ulcers, tuberculosis, emphysema/bronchitis, asthma and chronic obstructive pulmonary disease (participants with prior cancer were not included in this study).

Table S4. Baseline characteristics of participants by body fat percentage quintiles*

	Body fat percentage (sex-specific) quintiles					
	**Q1**	**Q2**	**Q3**	**Q4**	**Q5**	**All participants**
Number of participants	101,325	100,620	102,746	102,190	102,767	509,648
Body fat % (adjusted)	19.1 ± 5.1	24.1 ± 4.9	27.6 ± 5.1	31.3 ± 5.2	37.5 ± 6.8	27.9 ± 8.4
**Socio-demographic factors**						
Age, year	53.1 ± 11.6	51.5 ± 10.7	51.6 ± 10.5	51.6 ± 10.2	51.5 ± 10.2	52.0 ± 10.7
Female, %	61.0	59.2	57.9	59.8	59.4	59.0
Urban, %	32.6	40.1	46.2	50.2	51.2	44.1
High school education or above, %	20.2	21.2	21.6	21.4	20.4	21.0
Household income ≥20,000 yuan/year, %	39.4	41.8	43.3	44.3	44.5	42.7
**Lifestyle factors †**						
Ever-regular smokers, %	34.2	33.0	31.8	31.5	31.7	32.4
Ever-regular alcohol drinkers, %	17.1	18.4	19.0	19.5	20.4	19.0
Total physical activity, MET-hr/day	22.1 ± 14.1	22.0 ± 14.2	21.3 ± 14.0	20.6 ± 13.6	19.6 ± 13.2	21.1 ± 13.9
**Regular dietary intake, % ‡**						
Fresh fruits	25.5	27.6	28.7	29.4	29.5	28.2
Fresh vegetables	98.1	98.2	98.5	98.5	98.4	98.3
Meat	43.7	46.1	47.5	48.9	50.1	47.2
**Medical history, %**						
Has family history of cancer	16.1	16.7	17.1	17.5	17.7	17.0
Had any prior chronic diseases §	22.9	21.1	21.4	22.2	24.2	22.4

Abbreviations: MET-hr/day=metabolic equivalents of task hours per day.

* Plus–minus values are means ±SD.

Data were directly standardised to the age, sex, and region structure of the study population when appropriate.

† Ever-regular smokers include current- and ex-regular smokers; ever-regular alcohol drinkers include current-regular, ex-regular, and reduced-intake drinkers.

‡ Regular dietary intake refers to consumption on ≥4 days/week.

§ Chronic diseases included diabetes, chronic heart diseases, stroke/transient ischemic attack, chronic hepatitis/liver cirrhosis, kidney diseases, peptic ulcers, tuberculosis, emphysema/bronchitis, asthma and chronic obstructive pulmonary disease (participants with prior cancer were not included in this study).

Table S5. Pearson partial correlations between baseline body composition measures*

		Standing height	Weight	BMI	WC	HC	WHR	BF%	Fat mass	Fat-free mass	BMI25
**Standing height**	ALL	1.00	0.49	0.04	0.22	0.39	-0.04	0.00	0.21	0.69	-0.17
	M	1.00	0.52	0.07	0.27	0.43	0.01	0.05	0.24	0.67	-0.19
	F	1.00	0.46	0.01	0.18	0.36	-0.07	-0.02	0.19	0.74	-0.15
**Weight**	ALL		1.00	0.88	0.84	0.84	0.48	0.74	0.89	0.80	0.20
	M		1.00	0.88	0.86	0.85	0.53	0.70	0.87	0.87	0.16
	F		1.00	0.89	0.82	0.84	0.43	0.77	0.92	0.74	0.22
**BMI**	ALL			1.00	0.85	0.77	0.56	0.86	0.92	0.54	0.31
	M			1.00	0.86	0.76	0.62	0.80	0.89	0.65	0.29
	F			1.00	0.84	0.77	0.52	0.89	0.94	0.45	0.33
**WC**	ALL				1.00	0.77	0.78	0.77	0.84	0.56	0.18
	M				1.00	0.81	0.80	0.75	0.84	0.66	0.14
	F				1.00	0.75	0.77	0.78	0.84	0.47	0.22
**HC**	ALL					1.00	0.22	0.66	0.78	0.64	0.14
	M					1.00	0.30	0.63	0.76	0.72	0.10
	F					1.00	0.16	0.68	0.79	0.59	0.16
**WHR**	ALL						1.00	0.54	0.53	0.24	0.15
	M						1.00	0.57	0.58	0.33	0.12
	F						1.00	0.51	0.49	0.15	0.17
**BF%**	ALL							1.00	0.95	0.21	0.20
	M							1.00	0.95	0.27	0.13
	F							1.00	0.95	0.17	0.24
**Fat mass**	ALL								1.00	0.45	0.21
	M								1.00	0.52	0.16
	F								1.00	0.41	0.24
**Fat-free mass**	ALL									1.00	0.11
	M									1.00	0.12
	F									1.00	0.10
**BMI25**	ALL										1.00
	M										1.00
	F										1.00

Abbreviations: BMI=body mass index; WC=waist circumference; HC=hip circumference; WHR=waist-to-hip ratio; BF%=body fat percentage; BMI25=BMI at age 25; M=male; F=female.

* Adjusted for age, sex, and study areas, where appropriate.

Table S6. Adjusted HRs (95% CIs) for GI-cancer subtype/ subsites per SD higher usual levels of adiposity*

	ESCC		Cardia SC		Non-cardia SC †		Heterogeneity	Colon cancer		Rectal cancer		Heterogeneity
	Cases	HR (95%CI)	Cases	HR (95%CI)	Cases	HR (95%CI)	by SC subsite	Cases	HR (95%CI)	Cases	HR (95%CI)	by CRC subsite
**BMI**	726	0.86 (0.78 - 0.93)	388	1.02 (0.91 - 1.14)	2957	0.87 (0.83 - 0.91)	X^2 =6.4 (p=0.01)	1548	1.12 (1.06 - 1.18)	1511	1.08 (1.02 - 1.15)	X^2 =0.7 (p=0.4)
**BF%**	726	0.80 (0.72 - 0.90)	388	0.93 (0.80 - 1.08)	2957	0.77 (0.73 - 0.82)	X^2 =4.7 (p=0.03)	1548	1.10 (1.02 - 1.19)	1511	1.08 (1.00 - 1.17)	X^2 =0.2 (p=0.7)
**Fat mass**	726	0.84 (0.76 - 0.94)	388	0.98 (0.86 - 1.13)	2957	0.82 (0.78 - 0.86)	X^2 =5.8 (p=0.02)	1548	1.12 (1.05 - 1.20)	1511	1.09 (1.02 - 1.17)	X^2 =0.4 (p=0.5)
**Fat-free mass**	726	1.06 (0.97 - 1.15)	388	1.12 (0.99 - 1.26)	2957	0.97 (0.93 - 1.02)	X^2 =4.5 (p=0.03)	1548	1.11 (1.05 - 1.18)	1511	1.07 (1.00 - 1.13)	X^2 =0.8 (p=0.4)
**WC**	726	0.96 (0.87 - 1.05)	388	1.01 (0.89 - 1.15)	2957	0.86 (0.83 - 0.91)	X^2 =5.2 (p=0.02)	1548	1.21 (1.13 - 1.28)	1511	1.14 (1.07 - 1.21)	X^2 =1.7 (p=0.2)
**WHR**	726	1.00 (0.89 - 1.13)	388	0.94 (0.80 - 1.10)	2957	0.90 (0.85 - 0.95)	X^2 =0.2 (p=0.6)	1548	1.19 (1.11 - 1.28)	1511	1.17 (1.08 - 1.26)	X^2 =0.2 (p=0.6)
**BMI25**	625	1.06 (0.95 - 1.18)	336	1.04 (0.89 - 1.20)	2445	1.10 (1.04 - 1.16)	X^2 =0.5 (p=0.5)	1336	1.02 (0.95 - 1.10)	1257	1.10 (1.02 - 1.19)	X^2 =1.9 (p=0.2)

Abbreviations: ESCC=oesophageal squamous cell carcinoma; SC=stomach cancer; BMI=body mass index; BF%=body fat percentage; WC=waist circumference; WHR=waist-to-hip ratio; BMI25=BMI at age 25.

* Analyses were stratified by age-at-risk (10-year bands) and sex, and adjusted for 10 study areas, education level, household income level, family history of cancer, smoking, alcohol consumption, physical activity, and dietary factors significantly associated with the outcome being studied (i.e. fruits, preserved vegetables, soy and spicy food for EC, and fruits for SC). Sex-specific SDs were used.

† The study outcome definition for non-cardia SC (ICD-10 C16.1-C16.9) included unspecified (C16.9) cases, and preliminary event adjudication data (amongst a subset of SC cases) showed that the majority were confirmed to be C16.1-C16.6.

Table S7. Adjusted HRs (95% CIs) for oesophageal cancer per SD higher usual levels of adiposity, stratified by high-risk and non-high-risk areas*

	High-risk (Huixian)		Non-high-risk areas		Heterogeneity
	EC cases	HR (95%CI)	EC cases	HR (95%CI)	
**BMI**	1,188	0.82 (0.77 - 0.88)	1,162	0.79 (0.73 - 0.85)	X^2=0.8 (p=0.4)
**BF%**	1,188	0.75 (0.69 - 0.82)	1,162	0.76 (0.70 - 0.84)	X^2=0.1 (p=0.7)
**Fat mass**	1,188	0.79 (0.73 - 0.86)	1,162	0.75 (0.68 - 0.82)	X^2=0.8 (p=0.4)
**Fat-free mass**	1,188	0.99 (0.93 - 1.06)	1,162	0.87 (0.81 - 0.94)	X^2=6.6 (p=0.01)
**WC**	1,188	0.87 (0.81 - 0.94)	1,162	0.86 (0.80 - 0.93)	X^2<0.1 (p=0.9)
**WHR**	1,188	0.89 (0.81 - 0.97)	1,162	0.94 (0.86 - 1.03)	X^2=0.7 (p=0.4)
**BMI25**	1,056	1.02 (0.94 - 1.12)	948	1.14 (1.05 - 1.25)	X^2=3.1 (p=0.08)

Abbreviations: HR=hazard ratio; CI=confidence interval; SD = standard deviation; EC=oesophageal cancer; BMI=body mass index; BF%=body fat percentage; WC=waist circumference; WHR=waist-to-hip ratio; BMI25=BMI at age 25.

*Analyses were stratified by age-at-risk (10-year bands) and sex, and adjusted for study areas (where appropriate), education level, household income level, family history of cancer, smoking, alcohol consumption, physical activity, and intake of fruits, soy, preserved vegetables and spicy food. Sex-specific SDs were used.

Table S8. Associations of usual adiposity with GI-cancers (stratified by 5-year age-at-risk, sex, 10 study areas)

	EC		SC		CRC	
	**Cases**	**HR (95%CI)**	**Cases**	**HR (95%CI)**	**Cases**	**HR (95%CI)**
**BMI**						
per 5 kg/m²	2350	0.73 (0.68 - 0.79)	3345	0.84 (0.79 - 0.89)	3059	1.15 (1.09 - 1.23)
per SD	2350	0.81 (0.77 - 0.85)	3345	0.89 (0.85 - 0.93)	3059	1.10 (1.06 - 1.15)
**BF%**						
per 10%	2350	0.67 (0.60 - 0.73)	3345	0.71 (0.65 - 0.77)	3059	1.14 (1.05 - 1.24)
per SD	2350	0.76 (0.72 - 0.81)	3345	0.80 (0.76 - 0.84)	3059	1.10 (1.04 - 1.16)
**Fat mass**						
per 5kg	2350	0.83 (0.79 - 0.87)	3345	0.88 (0.84 - 0.91)	3059	1.08 (1.04 - 1.12)
per SD	2350	0.78 (0.73 - 0.83)	3345	0.85 (0.80 - 0.89)	3059	1.11 (1.06 - 1.17)
**Fat-free mass**						
per 5kg	2350	0.95 (0.90 - 0.99)	3345	0.99 (0.96 - 1.03)	3059	1.09 (1.05 - 1.13)
per SD	2350	0.94 (0.90 - 0.99)	3345	1.00 (0.96 - 1.04)	3059	1.09 (1.05 - 1.14)
**WC**						
per 10cm	2350	0.87 (0.82 - 0.92)	3345	0.88 (0.84 - 0.92)	3059	1.17 (1.12 - 1.23)
per SD	2350	0.87 (0.83 - 0.92)	3345	0.89 (0.85 - 0.93)	3059	1.17 (1.12 - 1.22)
**WHR**						
per SD	2350	0.91 (0.85 - 0.97)	3345	0.91 (0.86 - 0.96)	3059	1.17 (1.11 - 1.23)
**BMI25**						
per 5 kg/m²	2004	1.16 (1.03 - 1.31)	2781	1.17 (1.05 - 1.29)	2593	1.11 (1.00 - 1.23)
per SD	2004	1.08 (1.02 - 1.15)	2781	1.09 (1.03 - 1.14)	2593	1.06 (1.00 - 1.12)

Abbreviations: EC=oesophageal cancer; SC=stomach cancer; CRC=colorectal cancer; HR=hazard ratio; CI=confidence interval; SD=standard deviation; BMI=body mass index; BF%=body fat percentage; WC=waist circumference; WHR=waist-to-hip ratio; BMI25=BMI at age 25.

* Analyses were stratified by age-at-risk (5-year bands), sex, and 10 study areas, and adjusted for education level, household income level, family history of cancer, smoking, alcohol consumption, physical activity, and dietary factors (fruits, preserved vegetables, soy and spicy food for EC; and fruits for SC). Sex-specific SDs were used.

Table S9. Adjusted HRs (95% CIs) for GI-cancers per SD higher usual levels of adiposity, excluding the first 3 or 5 years of follow-up, any prior chronic diseases and self-rated poor health

	BMI		BF%		Fat mass		Fat-free mass		WC		WHR	
	N	HR (95% CI)	N	HR (95% CI)	N	HR (95% CI)	N	HR (95% CI)	N	HR (95% CI)	N	HR (95% CI)
**Oesophageal Cancer**												
Main model	2350	0.81 (0.77 - 0.85)	2350	0.76 (0.72 – 0.81)	2350	0.78 (0.73 – 0.83)	2350	0.93 (0.89 – 0.98)	2350	0.87 (0.83 – 0.92)	2350	0.91 (0.85 – 0.97)
Exclusion of first 3 yrs. of follow-up	1710	0.84 (0.80 - 0.89)	1710	0.82 (0.76 - 0.88)	1710	0.82 (0.77 - 0.88)	1710	0.94 (0.89 - 1.00)	1710	0.90 (0.85 - 0.96)	1710	0.95 (0.88 - 1.03)
Plus exclusion of any prior chronic diseases *	1236	0.86 (0.81 - 0.92)	1236	0.84 (0.77 - 0.92)	1236	0.85 (0.78 - 0.92)	1236	0.95 (0.88 - 1.02)	1236	0.93 (0.87 - 1.00)	1236	0.98 (0.89 - 1.08)
Plus exclusion of self-rated poor health	1113	0.87 (0.81 - 0.94)	1113	0.85 (0.78 - 0.94)	1113	0.86 (0.79 - 0.94)	1113	0.95 (0.88 - 1.02)	1113	0.95 (0.88 - 1.03)	1113	1.01 (0.91 - 1.11)
Exclusion of first 5 yrs. of follow-up only	1227	0.85 (0.80 - 0.91)	1227	0.81 (0.75 - 0.89)	1227	0.83 (0.76 - 0.90)	1227	0.97 (0.90 - 1.04)	1227	0.91 (0.85 - 0.98)	1227	0.96 (0.88 - 1.05)
**Stomach Cancer**												
Main model	3345	0.88 (0.85 – 0.92)	3345	0.79 (0.75 – 0.83)	3345	0.83 (0.79 – 0.88)	3345	0.99 (0.95 – 1.03)	3345	0.88 (0.84 – 0.92)	3345	0.90 (0.86 – 0.95)
Exclusion of first 3 yrs. of follow-up	2423	0.93 (0.89 - 0.97)	2423	0.87 (0.81 - 0.92)	2423	0.90 (0.85 - 0.95)	2423	1.00 (0.95 - 1.05)	2423	0.94 (0.89 - 0.99)	2423	0.97 (0.91 - 1.04)
Plus exclusion of any prior chronic diseases *	1710	0.93 (0.88 - 0.98)	1710	0.87 (0.81 - 0.94)	1710	0.90 (0.84 - 0.97)	1710	0.99 (0.93 - 1.05)	1710	0.94 (0.88 - 1.00)	1710	0.99 (0.92 - 1.07)
Plus exclusion of self-rated poor health	1561	0.94 (0.88 - 0.99)	1561	0.88 (0.81 - 0.95)	1561	0.91 (0.85 - 0.98)	1561	0.98 (0.92 - 1.04)	1561	0.94 (0.88 - 1.00)	1561	0.98 (0.90 - 1.06)
Exclusion of first 5 yrs. of follow-up only	1828	0.91 (0.86 - 0.96)	1828	0.84 (0.79 - 0.91)	1828	0.88 (0.83 - 0.94)	1828	1.00 (0.94 - 1.05)	1828	0.93 (0.88 - 0.99)	1828	0.96 (0.89 - 1.03)
**Colorectal Cancer**												
Main model	3059	1.10 (1.06 – 1.15)	3059	1.09 (1.03 – 1.15)	3059	1.11 (1.05 – 1.16)	3059	1.09 (1.04 – 1.14)	3059	1.17 (1.12 – 1.22)	3059	1.18 (1.12 – 1.24)
Exclusion of first 3 yrs. of follow-up	2417	1.14 (1.09 - 1.20)	2417	1.15 (1.08 - 1.22)	2417	1.15 (1.09 - 1.21)	2417	1.10 (1.05 - 1.16)	2417	1.21 (1.15 - 1.28)	2417	1.21 (1.14 - 1.28)
Plus exclusion of any prior chronic diseases *	1673	1.13 (1.07 - 1.19)	1673	1.13 (1.05 - 1.22)	1673	1.13 (1.06 - 1.21)	1673	1.08 (1.02 - 1.15)	1673	1.20 (1.13 - 1.27)	1673	1.22 (1.14 - 1.30)
Plus exclusion of self-rated poor health	1562	1.14 (1.07 - 1.20)	1562	1.13 (1.05 - 1.22)	1562	1.13 (1.06 - 1.21)	1562	1.09 (1.03 - 1.16)	1562	1.21 (1.14 - 1.29)	1562	1.22 (1.15 - 1.30)
Exclusion of first 5 yrs. of follow-up only	1824	1.17 (1.11 - 1.23)	1824	1.18 (1.11 - 1.27)	1824	1.19 (1.12 - 1.26)	1824	1.13 (1.07 - 1.20)	1824	1.27 (1.20 - 1.34)	1824	1.24 (1.18 - 1.31)

Abbreviations: HR=hazard ratio; CI=confidence interval; SD=standard deviation; BMI=body mass index; BF%=body fat percentage; WC=waist circumference; WHR=waist-to-hip ratio.

Analyses were stratified by age-at-risk (10-year bands) and sex, and adjusted for 10 study areas, education level, household income level, family history of cancer, smoking, alcohol consumption, physical activity and dietary factors (fruit, soy, preserved vegetables and spicy food for EC; and fruit for SC). Sex-specific SDs were used.

*Chronic diseases included diabetes, chronic heart diseases, stroke/transient ischemic attack, chronic hepatitis/liver cirrhosis, kidney diseases, peptic ulcers, tuberculosis, emphysema/bronchitis, asthma and chronic obstructive pulmonary disease (participants with prior cancer were not included in this study)

Figure S1. Adjusted means of selected baseline adiposity traits by CKB study areas

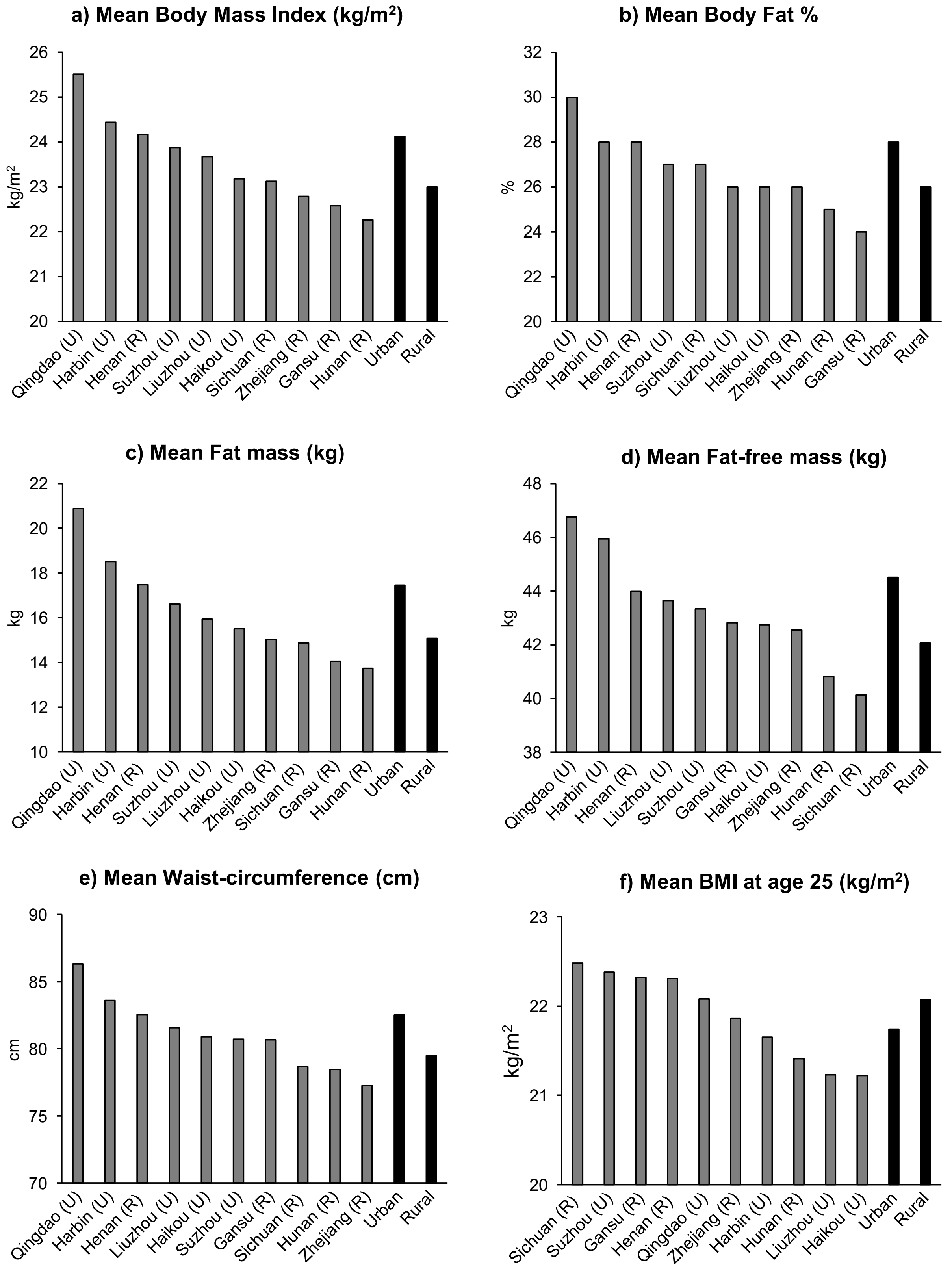

*Means were adjusted for age and sex.

Figure S2. Adjusted HRs for GI-cancers by usual levels of BMI: A) WHO-international cut-offs, B) WHO-Asian cut-offs, and C) CKB cut-offs

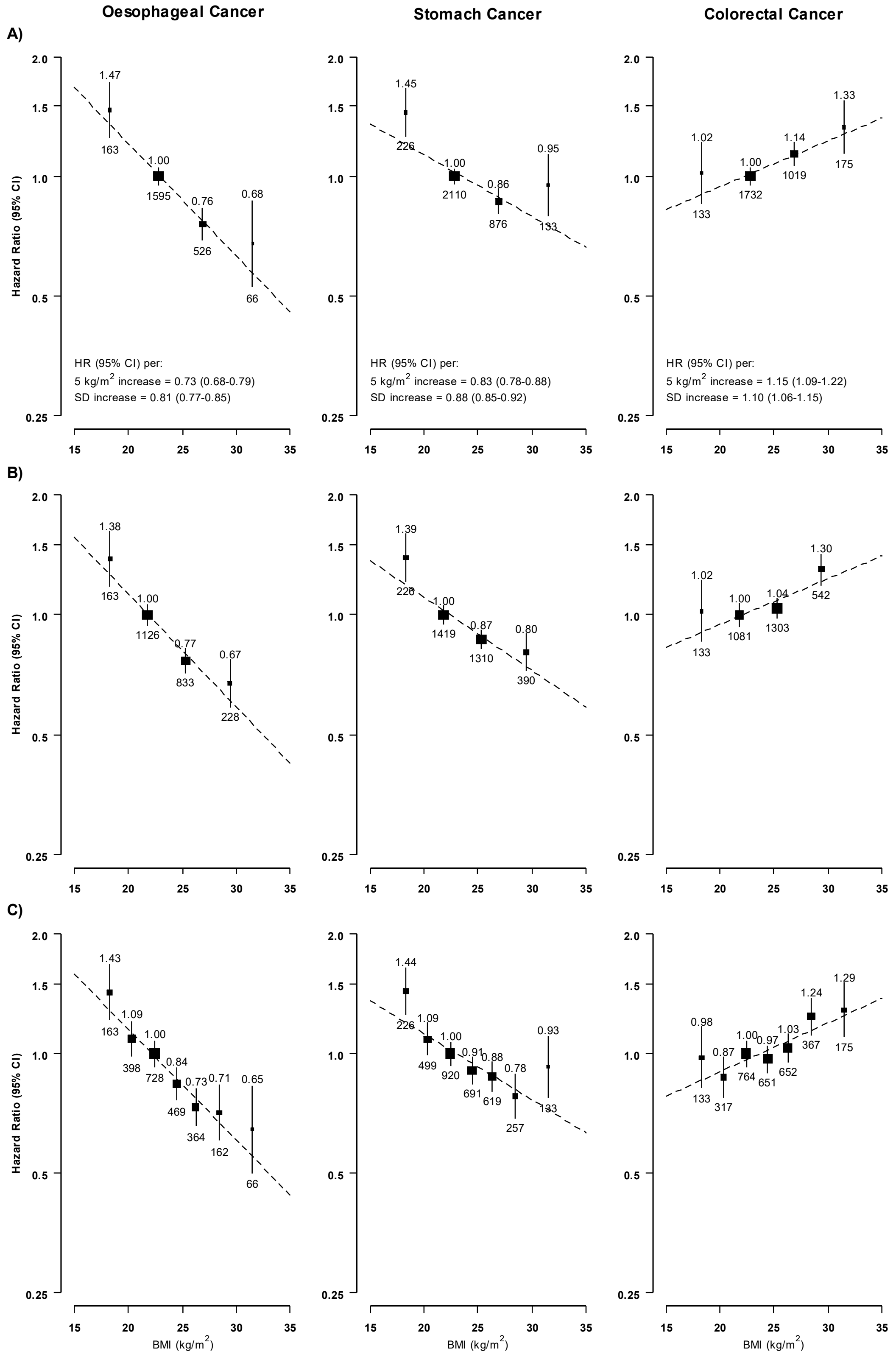

Abbreviations: WHO=World Health Organization; HR=hazard ratio; CI=confidence interval; SD=standard deviation; BMI=body mass index. Analyses were stratified by age-at-risk (10-year bands) and sex, and adjusted for 10 study areas, education level, household income level, family history of cancer, smoking, alcohol consumption, physical activity and dietary factors (fruit, soy, preserved vegetables and spicy food for EC; and fruit for SC). HRs were plotted against the mean values at second resurvey in baseline-defined categories. Vertical lines represent floated 95%CIs. The area of each square is inversely proportional to the variance of the log HR. Numbers above the squares are HRs and numbers below are number of events. Sex-specific SDs were used.

Figure S3. Associations of baseline fat and fat-free mass with GI-cancers, with mutual adjustments

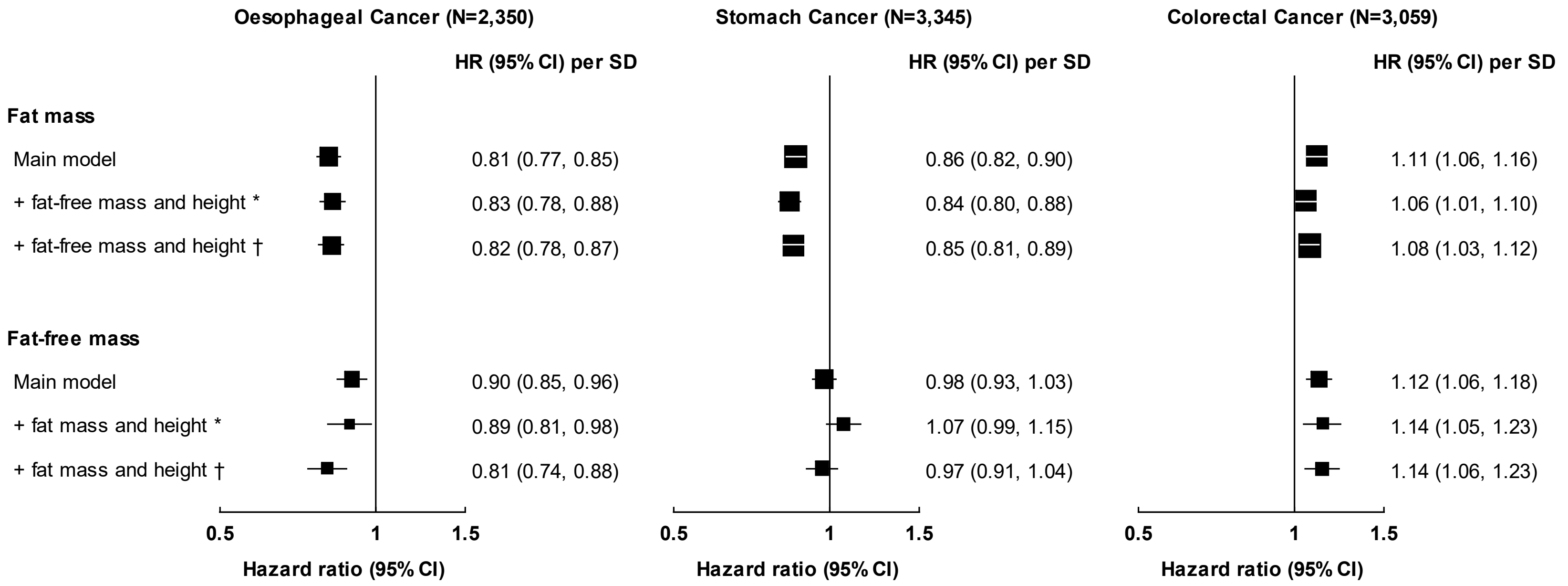

Abbreviations: HR=hazard ratio; CI=confidence interval; SD=standard deviation. HRs were not corrected for regression dilution bias and overall SDs were used, to avoid complications in interpretation when making comparisons with the residuals model. The main models were stratified by age-at-risk (10-year bands) and sex, and adjusted for 10 study areas, education level, household income level, family history of cancer, smoking, alcohol consumption, physical activity and dietary factors (fruit, soy, preserved vegetables and spicy food for EC; and fruit for SC). The area of each square is inversely proportional to the variance of the log HR.

* “Conventional” adjustment (i.e., included as covariates in the model)

† Residuals method

Figure S4. Comparison across adiposity traits

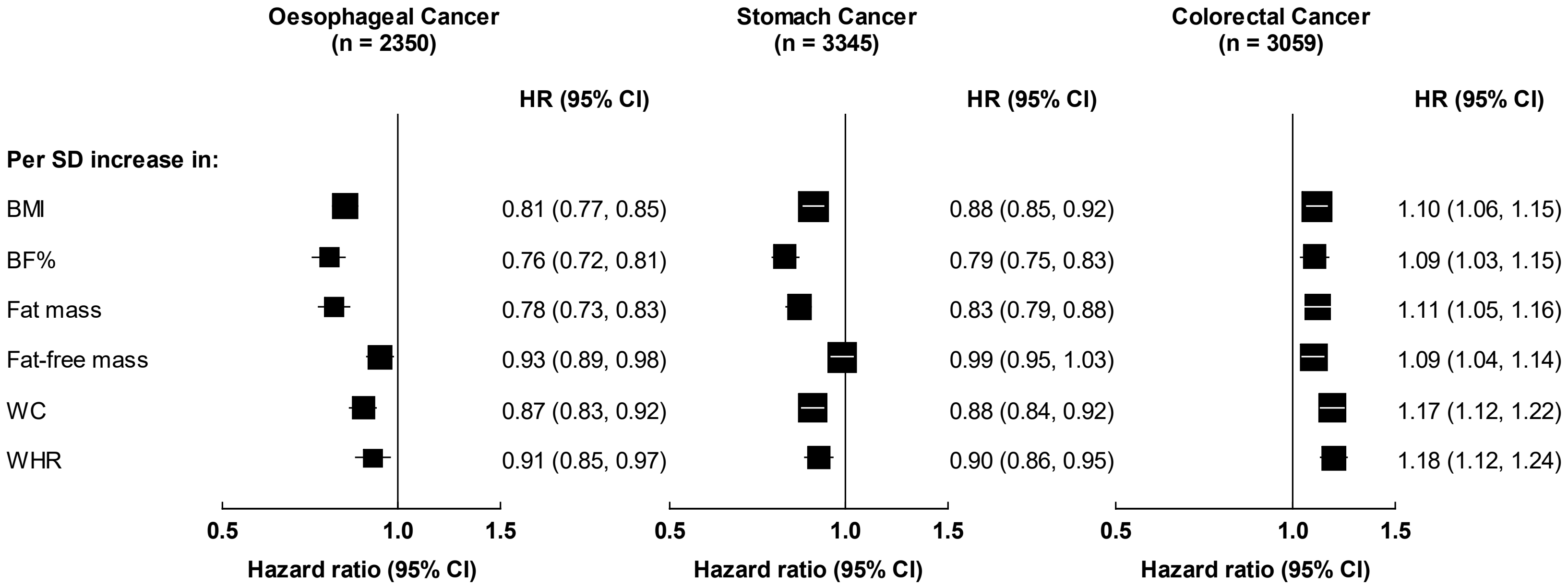

Abbreviations: HR=hazard ratio; CI=confidence interval; SD=standard deviation; BMI=body mass index; BF%=body fat percentage; WC=waist circumference; WHR=waist-to-hip ratio.

Analyses were stratified by age-at-risk (10-year bands) and sex, and adjusted for 10 study areas, education level, household income level, family history of cancer, smoking, alcohol consumption, physical activity and dietary factors (fruit, soy, preserved vegetables and spicy food for EC; and fruit for SC). Sex-specific SDs were used.

Figure S5. Associations of baseline general and central adiposity with GI-cancers, with mutual adjustments

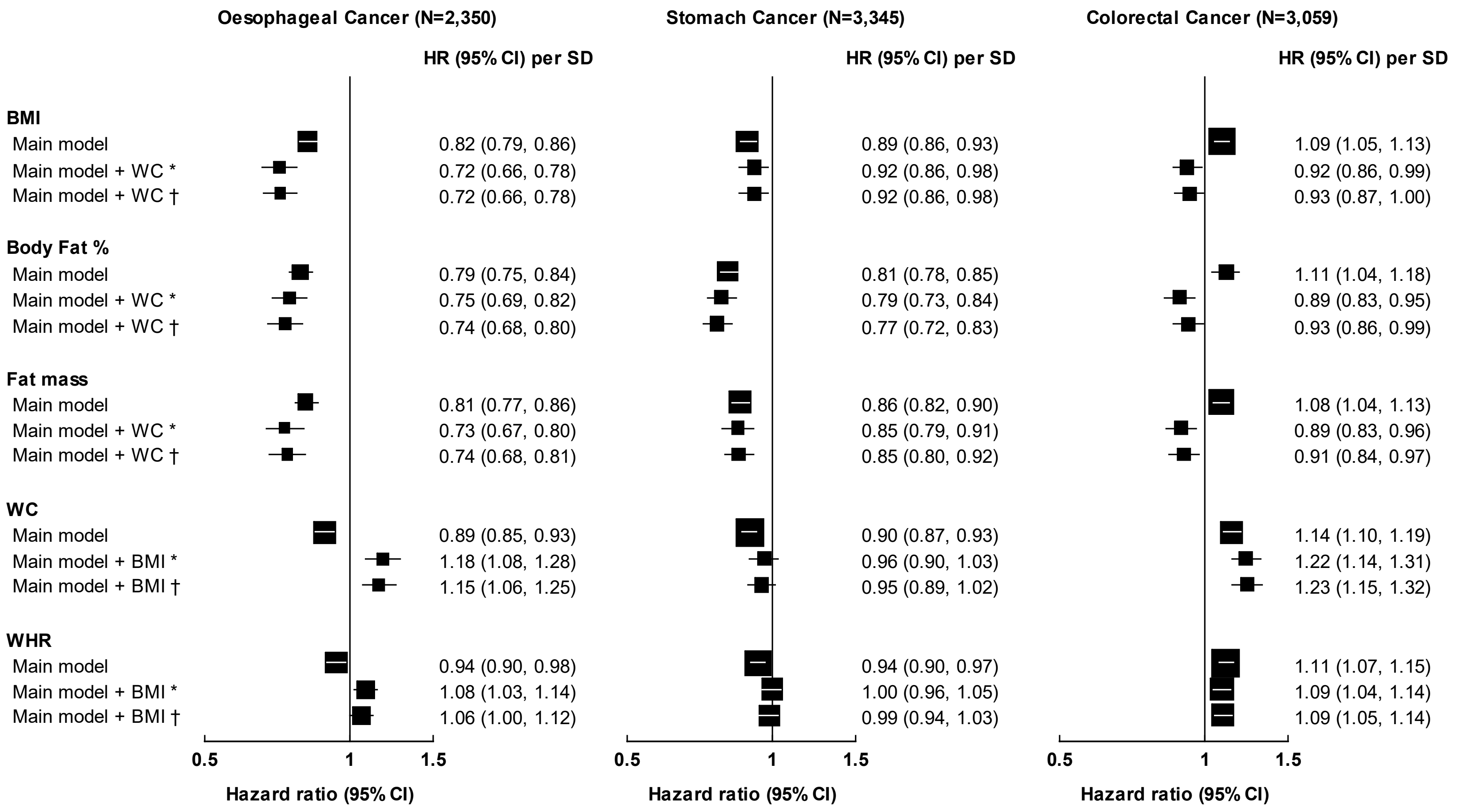

Abbreviations: HR=hazard ratios; CI=confidence interval; SD=standard deviation; BMI=body mass index; WC=waist circumference; WHR=waist-to-hip ratio.
HRs were not corrected for regression dilution bias and overall SDs were used, to avoid complications in interpretation when making comparisons with the residuals model. The main model was stratified by age-at-risk (10-year bands) and sex, and adjusted for 10 study areas, education level, household income level, family history of cancer, smoking, alcohol consumption, physical activity and dietary factors (fruit, soy, preserved vegetables and spicy food for EC; and fruit for SC).

* Adjusted as a covariate in the model. † Residuals method

Figure S6. Adjusted HRs for GI-cancers by usual BMI at age 25

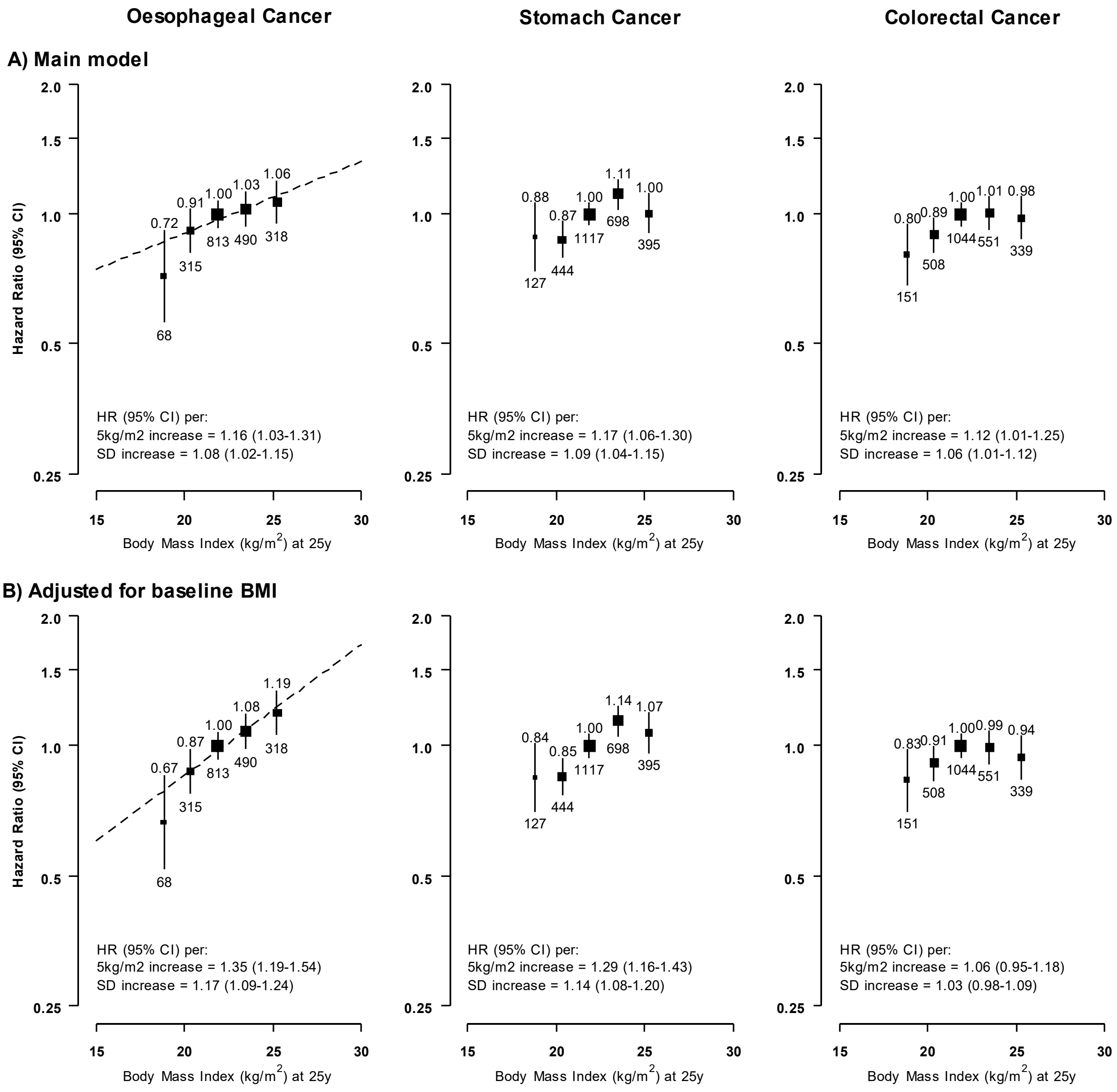

Conventions as per Figure S2. In the main model (A), analyses were stratified by age-at-risk (10-year bands) and sex, and adjusted for 10 study areas, education level, household income level, family history of cancer, smoking, alcohol consumption, physical activity and dietary factors (fruit, soy, preserved vegetables and spicy food for EC; and fruit for SC). In (B), analyses were further adjusted for baseline BMI.

Figure S7. Associations of all adiposity traits with risk of non-cardia SC

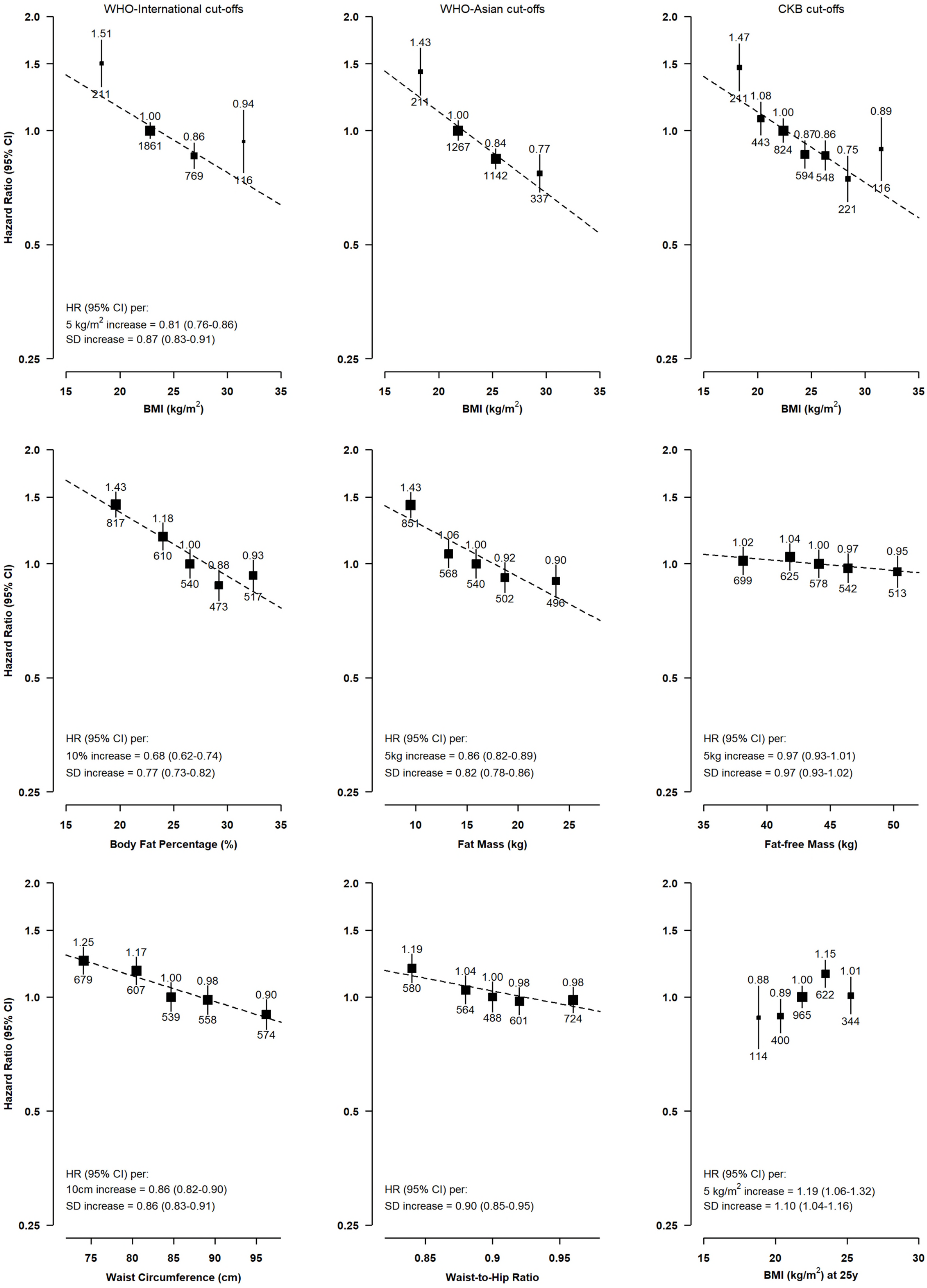

Conventions as per Figure S2. Associations remained statistically-significant (except fat-free mass) after exclusion of the first 3 years of follow-up (not applicable for the analysis of BMI25). The study outcome definition for non-cardia SC (ICD-10 C16.1-C16.9) included unspecified (C16.9) cases, and preliminary event adjudication data (amongst a subset of SC cases) showed that the majority were confirmed to be C16.1-C16.6.

Figure S8. Adjusted HRs for GI-cancers per SD increase in usual levels of BMI, by selected baseline characteristics

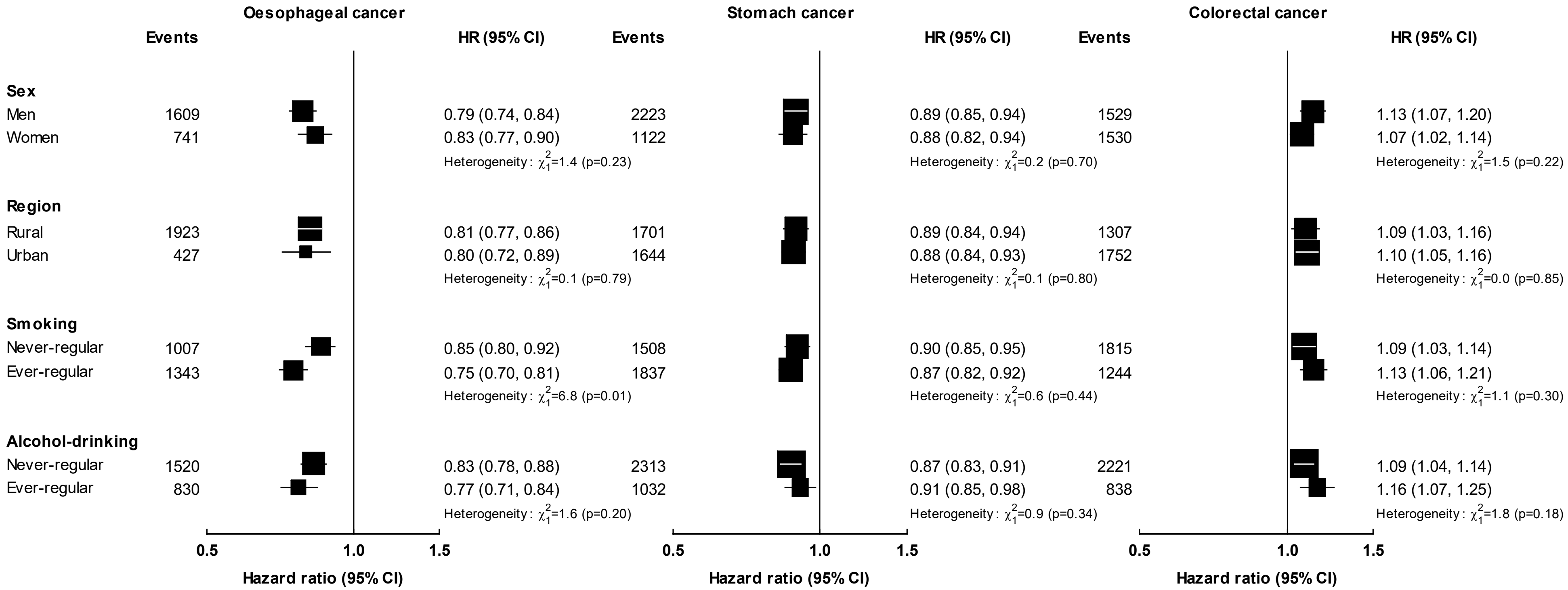

Abbreviations: SD=standard deviation; BMI=body mass index. Analyses were stratified by age-at-risk (10-year bands) and sex, adjusted for 10 study areas, education level, household income level, family history of cancer, smoking, alcohol consumption, physical activity and dietary factors (fruit, soy, preserved vegetables and spicy food for EC; and fruit for SC), where appropriate. Sex-specific SDs were used. The area of each square is inversely proportional to the variance of the log HR.

The HRs in individuals who never smoked or drank alcohol regularly are 0.86 (0.80-0.92) for EC, 0.89 (0.84-0.95) for SC, and 1.08 (1.03-1.14) for CRC, per SD increase in BMI.

Figure S9. Adjusted HRs for GI-cancers per SD increase in usual levels of body fat %, by selected baseline characteristics

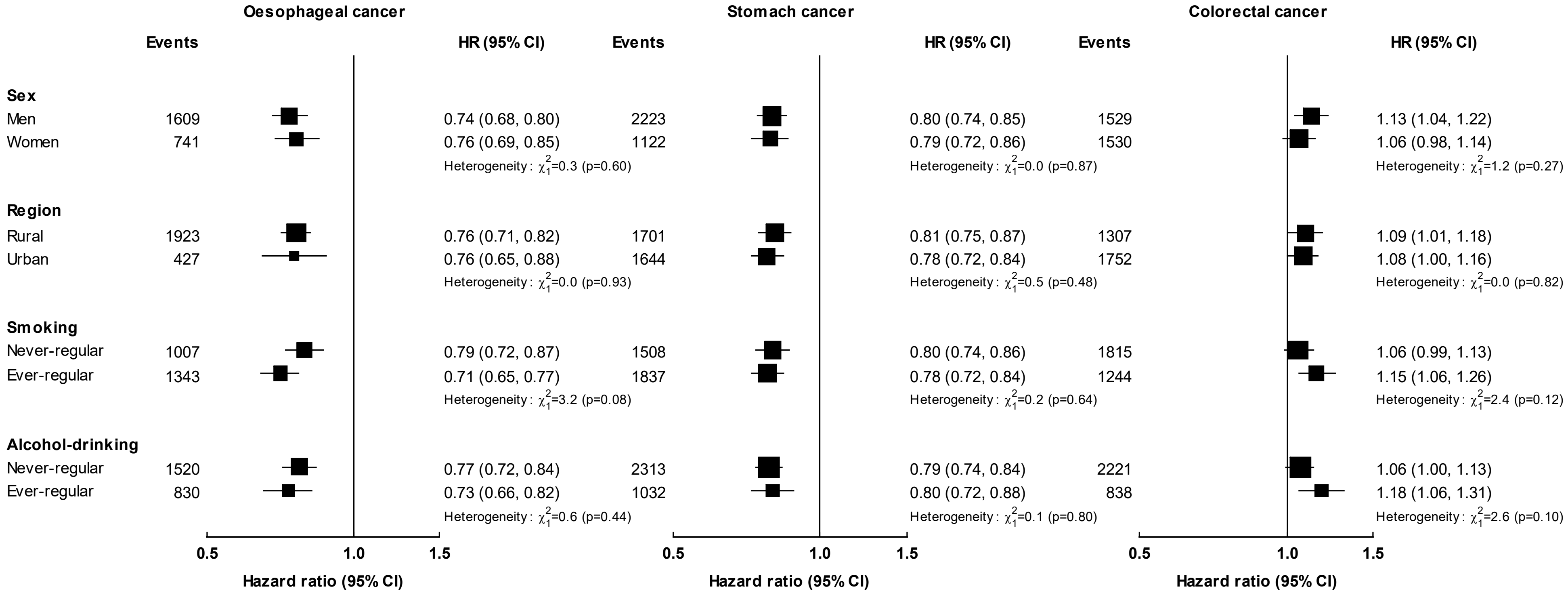

Conventions as per Figure S8.

Figure S10. Adjusted HRs for GI-cancers per SD increase in usual levels of fat mass, by selected baseline characteristics

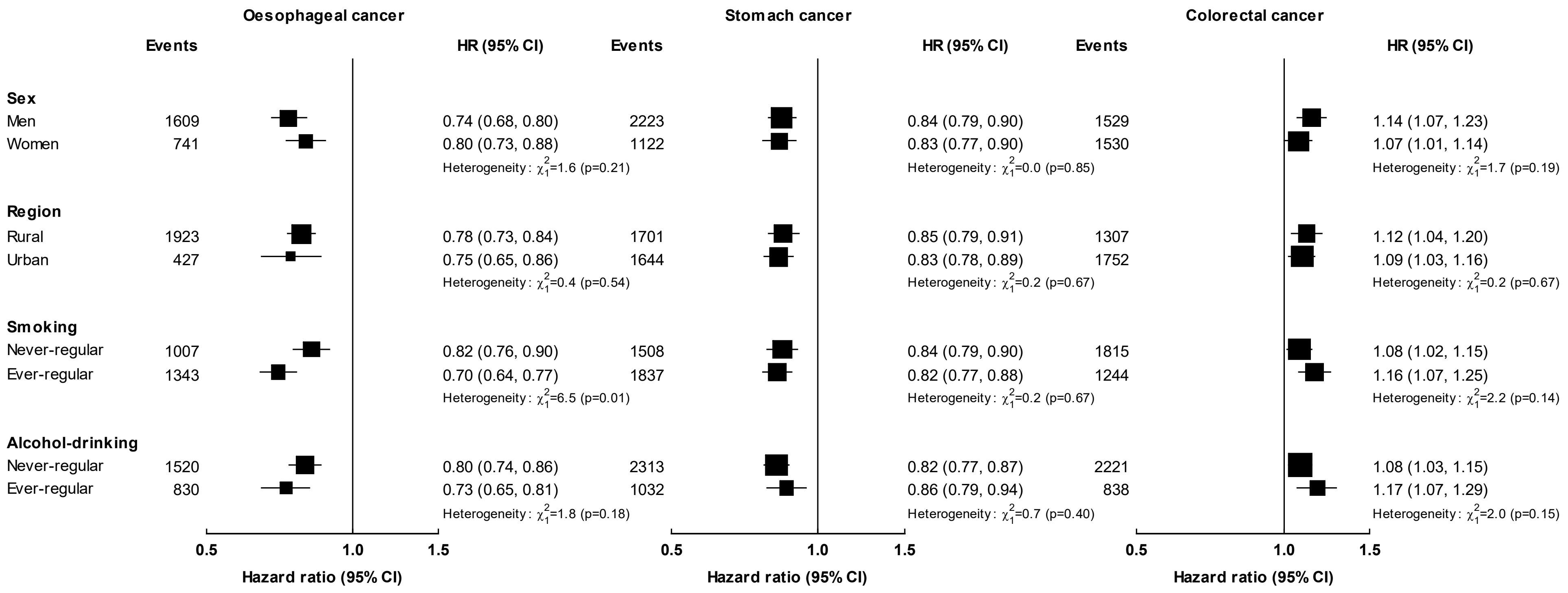

Conventions as per Figure S8.

Figure S11. Adjusted HRs for GI-cancers per SD increase in usual levels of fat-free mass, by selected baseline characteristics

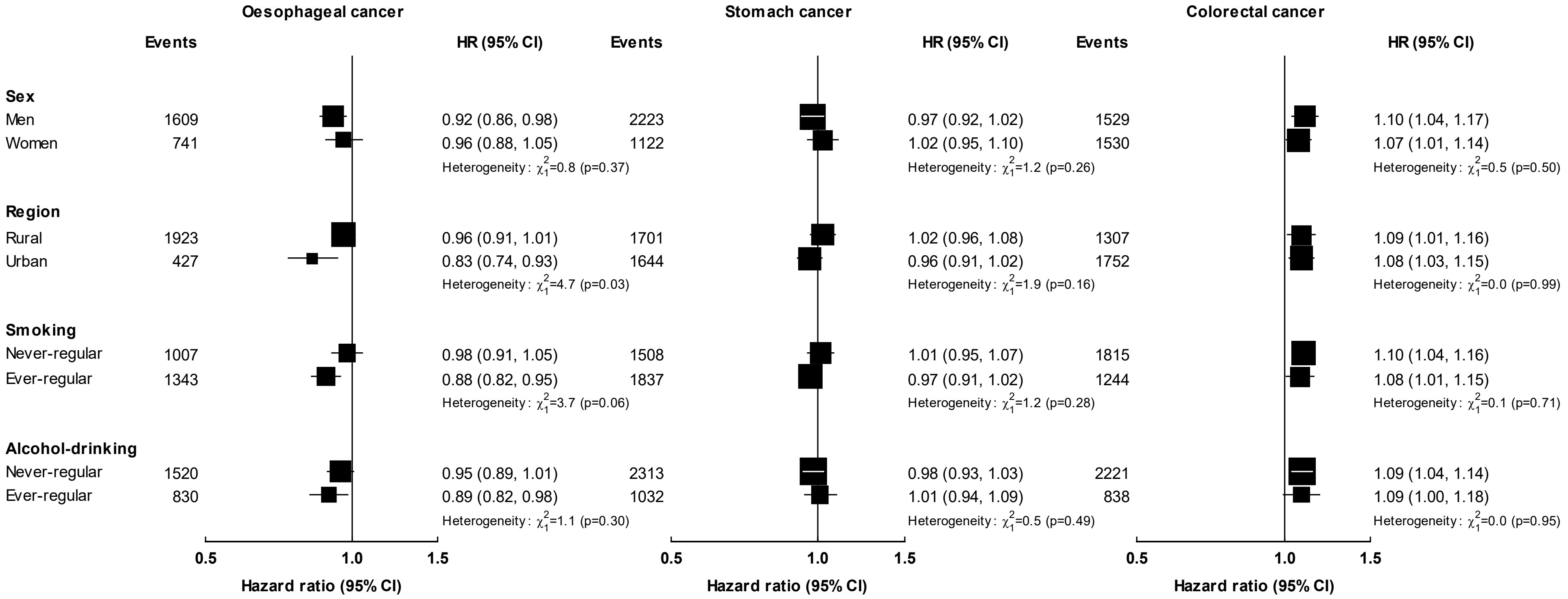

Conventions as per Figure S8.

Figure S12. Adjusted HRs for GI-cancers per SD increase in usual levels of waist circumference, by selected baseline characteristics

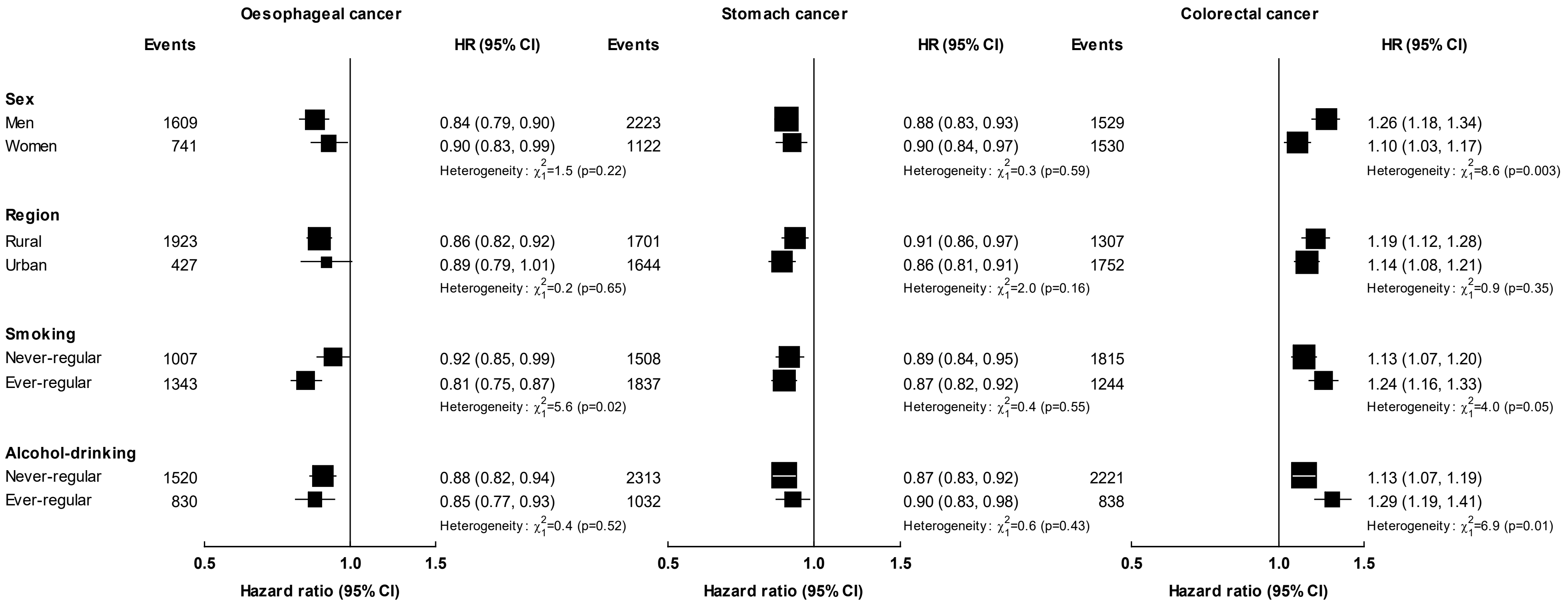

Conventions as per Figure S8.

Figure S13. Adjusted HRs for GI-cancers per SD increase in usual levels of WHR, by selected baseline characteristics

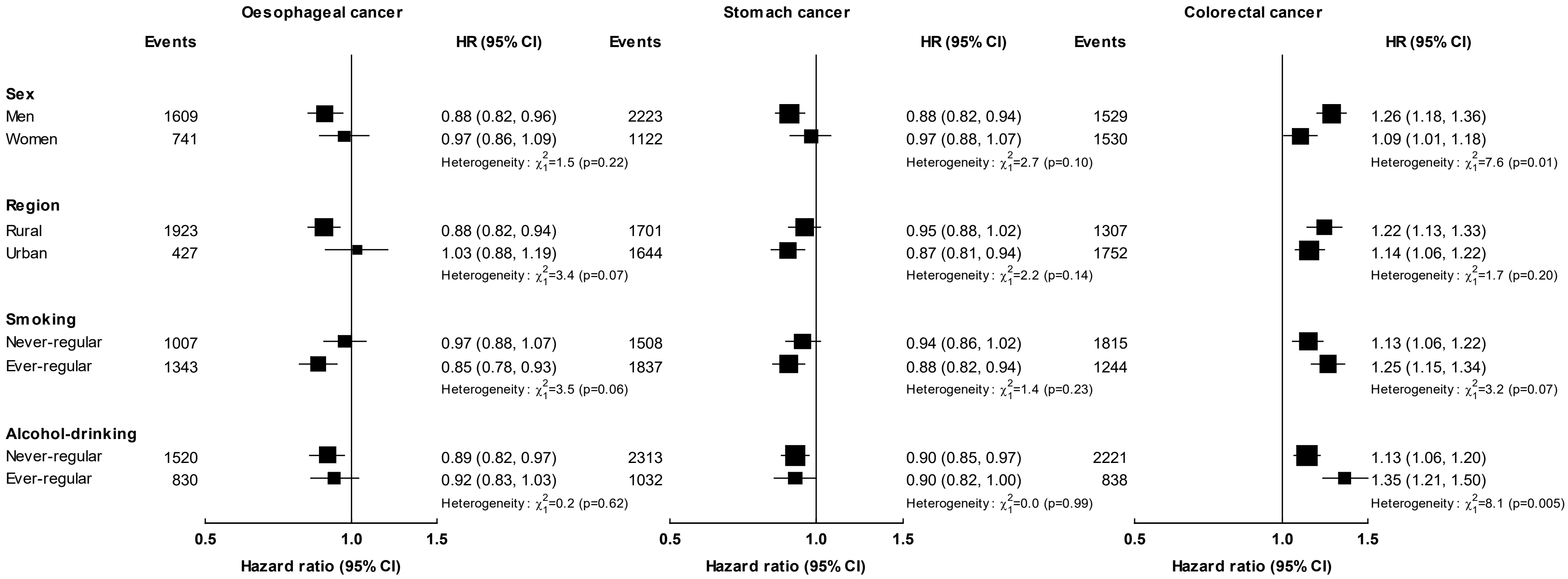

Abbreviations: WHR=waist-to-hip ratio, otherwise conventions as per Figure S8.

Members of the China Kadoorie Biobank collaborative group

International Steering Committee: Junshi Chen, Zhengming Chen (PI), Robert Clarke, Rory Collins, Liming Li (PI), Chen Wang, Jun Lv, Richard Peto, Robin Walters.

International Co-ordinating Centre, Oxford: Daniel Avery, Maxim Barnard, Derrick Bennett, Ruth Boxall, Ka Hung Chan, Yiping Chen, Zhengming Chen, Johnathan Clarke; Robert Clarke, Huaidong Du, Ahmed Edris Mohamed, Hannah Fry, Simon Gilbert, Pek Kei Im, Andri Iona, Maria Kakkoura, Christiana Kartsonaki, Hubert Lam, Kuang Lin, James Liu, Mohsen Mazidi, Iona Millwood, Sam Morris, Qunhua Nie, Alfred Pozarickij, Paul Ryder, Saredo Said, Dan Schmidt, Becky Stevens, Iain Turnbull, Robin Walters, Baihan Wang, Lin Wang, Neil Wright, Ling Yang, Xiaoming Yang, Pang Yao.

National Co-ordinating Centre, Beijing: Xiao Han, Can Hou, Qingmei Xia, Chao Liu, Jun Lv, Pei Pei, Dianjianyi Sun, Canqing Yu

10 Regional Co-ordinating Centres:

Guangxi Provincial CDC: Naying Chen, Duo Liu, Zhenzhu Tang. Liuzhou CDC: Ningyu Chen, Qilian Jiang, Jian Lan, Mingqiang Li, Yun Liu, Fanwen Meng, Jinhuai Meng, Rong Pan, Yulu Qin, Ping Wang, Sisi Wang, Liuping Wei, Liyuan Zhou. **Gansu** Provincial CDC: Caixia Dong, Pengfei Ge, Xiaolan Ren. Maiji CDC: Zhongxiao Li, Enke Mao, Tao Wang, Hui Zhang, Xi Zhang. **Hainan** Provincial CDC: Jinyan Chen, Ximin Hu, Xiaohuan Wang. Meilan CDC: Zhendong Guo, Huimei Li, Yilei Li, Min Weng, Shukuan Wu. **Heilongjiang** Provincial CDC: Shichun Yan, Mingyuan Zou, Xue Zhou. Nangang CDC: Ziyan Guo, Quan Kang, Yanjie Li, Bo Yu, Qinai Xu. **Henan** Provincial CDC: Liang Chang, Lei Fan, Shixian Feng, Ding Zhang, Gang Zhou. Huixian CDC: Yulian Gao, Tianyou He, Pan He, Chen Hu, Huarong Sun, Xukui Zhang. **Hunan** Provincial CDC: Biyun Chen, Zhongxi Fu, Yuelong Huang, Huilin Liu, Qiaohua Xu, Li Yin. Liuyang CDC: Huajun Long, Xin Xu, Hao Zhang, Libo Zhang. **Jiangsu** Provincial CDC: Jian Su, Ran Tao, Ming Wu, Jie Yang, Jinyi Zhou, Yonglin Zhou. Suzhou CDC: Yihe Hu, Yujie Hua, Jianrong Jin, Fang Liu, Jingchao Liu, Yan Lu, Liangcai Ma, Aiyu Tang, Jun Zhang. **Qingdao** CDC: Liang Cheng, Ranran Du, Ruqin Gao, Feifei Li, Shanpeng Li, Yongmei Liu, Feng Ning, Zengchang Pang, Xiaohui Sun, Xiaocao Tian, Shaojie Wang, Yaoming Zhai, Hua Zhang, Licang CDC: Wei Hou, Silu Lv, Junzheng Wang. **Sichuan** Provincial CDC: Xiaofang Chen, Xianping Wu, Ningmei Zhang, Xiaoyu Chang. Pengzhou CDC: Xiaofang Chen, Jianguo Li, Jiaqiu Liu, Guojin Luo, Qiang Sun, Xunfu Zhong. **Zhejiang** Provincial CDC: Weiwei Gong, Ruying Hu, Hao Wang,Meng Wang, Min Yu. Tongxiang CDC: Lingli Chen, Qijun Gu, Dongxia Pan, Chunmei Wang, Kaixu Xie, Xiaoyi Zhang.

References

1. Willett WC, Howe GR, Kushi LH. Adjustment for total energy intake in epidemiologic studies. *Am J Clin Nutr*. Apr 1997;65(4 Suppl):1220S-1228S; discussion 1229S-1231S. doi:10.1093/ajcn/65.4.1220S

2. Omiyale W, Allen NE, Sweetland S. Body size, body composition and endometrial cancer risk among postmenopausal women in UK Biobank. *Int J Cancer*. Nov 1 2020;147(9):2405-2415. doi:10.1002/ijc.33023

3. Sweetland S, Floud S, Gaitskell K, Reeves GK. Adiposity and risk of oesophageal cancer subtypes in the Million Women Study. *Int J Epidemiol*. Dec 25 2023;52(6):1795-1804. doi:10.1093/ije/dyad094

4. Chen Z, Iona A, Parish S, et al. Adiposity and risk of ischaemic and haemorrhagic stroke in 0.5 million Chinese men and women: a prospective cohort study. *The Lancet Global health*. Jun 2018;6(6):e630-e640. doi:10.1016/s2214-109x(18)30216-x
